# Comparative transcriptomic analysis reveals the molecular mechanism underlying seedling biomass heterosis in *Brassica napus*

**DOI:** 10.1186/s12870-022-03671-0

**Published:** 2022-06-09

**Authors:** Jie Xiong, Kaining Hu, Nesma Shalby, Chenjian Zhuo, Jing Wen, Bin Yi, Jinxiong Shen, Chaozhi Ma, Tingdong Fu, Jinxing Tu

**Affiliations:** National Key Laboratory of Crop Genetic Improvement, Hubei Hongshan Laboratory, Huazhong Agricultural University, Wuhan, 430070 China

**Keywords:** *Brassica napus*, Heterosis, Plant hormones, Cell size, Photosynthesis, RNA-seq

## Abstract

**Background:**

Heterosis is an important biological phenomenon in which the hybrids exceed the parents in many traits. However, the molecular mechanism underlying seedling heterosis remains unclear.

**Results:**

In the present study, we analyzed the leaf transcriptomes of strong hybrids (AM, HM) and weak hybrids (CM, HW) and their parents (A, C, H, M, and W) at two periods. Phenotypically, hybrids had obvious biomass heterosis at the seedling stage, with statistically significant differences between the strong and weak hybrids. The transcriptomic analysis demonstrated that the number of differentially expressed genes (DEGs) between parents was the highest. Further analysis showed that most DEGs were biased toward parental expression. The biological processes of the two periods were significantly enriched in the plant hormone signal transduction and photosynthetic pathways. In the plant hormone signaling pathway, DEG expression was high in hybrids, with expression differences between strong and weak hybrids. In addition, DEGs related to cell size were identified. Similar changes were observed during photosynthesis. The enhanced leaf area of hybrids generated an increase in photosynthetic products, which was consistent with the phenotype of the biomass. Weighted gene co-expression network analysis of different hybrids and parents revealed that hub genes in vigorous hybrid were mainly enriched in the plant hormone signal transduction and regulation of plant hormones.

**Conclusion:**

Plant hormone signaling and photosynthesis pathways, as well as differential expression of plant cell size-related genes, jointly regulate the dynamic changes between strong and weak hybrids and the generation of seedling-stage heterosis. This study may elucidate the molecular mechanism underlying early biomass heterosis and help enhance canola yield.

**Supplementary Information:**

The online version contains supplementary material available at 10.1186/s12870-022-03671-0.

## Background

Heterosis refers to the phenomenon that hybrids perform better than their parents in various traits, such as biomass and resistance [1], and it has been successfully applied in field crops. It is a complicated phenomenon that may be coordinated by one or numerous genes and has always attracted the attention of breeders and molecular biologists. In the past century, various hypotheses have been proposed, including the dominance hypothesis [2], overdominance hypothesis [3], and epistasis hypothesis [4]. These hypotheses are limited to explaining the genetic basis of heterosis at the DNA level but do not reveal the biological basis for its occurrence. Furthermore, the mechanism underlying phenotypic heterosis is not completely understood.

The rapid development of sequencing technology has made it possible to explore heterosis at the molecular level [5], and it has been used in many species, such as *Arabidopsis* [6–8], rice [9–11], maize [12, 13], and wheat [14, 15]. These studies combined morphological and omics techniques to analyze the changes in the vegetative and reproductive growth phases of hybrids relative to their parents. Nonetheless, these studies did not determine the changes in important genes that participated in the adjustment of the biological pathways of biomass heterosis.

The seedling stage is a critical period for plants to accumulate nutrients and grow, and the coordinated source-sink-flow relationship contributes to the formation of yield at the mature stage [16]. Researchers have focused on the molecular mechanisms underlying early heterosis [17–20]. Leaves are important photosynthetic organs and are critical for biomass heterosis [21]. Transcriptomic analyses of *Arabidopsis* hybrids and their parents showed that the increase in the expression of indole-3-acetic acid (IAA)-targeted genes and the decrease in the expression of salicylic acid biosynthetic pathway-related genes in the hybrids were consistent with the phenotype of increased leaf cell size [22]. Transcriptomic analyses of canola hybrids and their parents four and eight days after sowing (DAS) revealed that the early biomass heterosis of seedlings in hybrids was related to the photosynthetic pathway and auxin biosynthetic pathway genes [7]. Liu et al. studied the temporal dynamic transcriptome of Col-0 and Per-1 and their hybrid seedlings during early development and found that the dominant expression complementation of the basic biological pathways helps the formation of heterosis [20]. However, most studies only focus on the heterosis of one or two hybrids and their parents, ignoring the comparative study of multiple pairs of hybrids and parents at different time points.

The utilization of canola (*Brassica napus*) heterosis effectively enhances the yield of edible vegetable oil. Canola and the model plant genus *Arabidopsis* belong to the family Brassicaceae. *B. napus* is developed through an interspecific cross between *B. rapa* (AA) and *B. oleracea* (CC). It is a young crop that originated approximately 7,500 years ago [23]. Moreover, expression level dominance (ELD) has been widely reported in allopolyploid plants [14, 20]. Transcriptomic, small RNA, and methylation sequencing analyses have systematically revealed the epigenetic mechanism underlying the heterosis of yield traits in canola [24], research on canola (*B. napus*) heterosis is still limited [24, 24, 25].

In the present study, we analyzed the leaf transcriptomes of AM, CM, HM, and HW hybrids and their parents (A, C, H, M, and W) at 21 and 24 DAS. This study demonstrates that affinities and distinctions in gene expression changes occur between strong and weak hybrids and their parents in the early stages of seedling development. A comparative analysis of the whole genome transcriptome comparison will help us to better understand the mechanism underlying gene expression changes in the hybrid and provide new ideas for hybrid breeding in canola.

## Results

### Phenotypic analysis of hybrids and parents

To better compare the biomass heterosis between the different hybrids, we calculated the mid-parent heterosis (MPH) and high-parent heterosis (HPH) of traits and found that the MPH (83.77%) and HPH (68.75%) of the AM combination (dry weight) were significantly higher than those of the parents at 21 DAS (Table S1). The MPH and HPH ranges in the dry weight were 18.64 – 83.77% and 6.98 – 68.75%, respectively, at 21 DAS (Table S1).

**Fig. 1:**
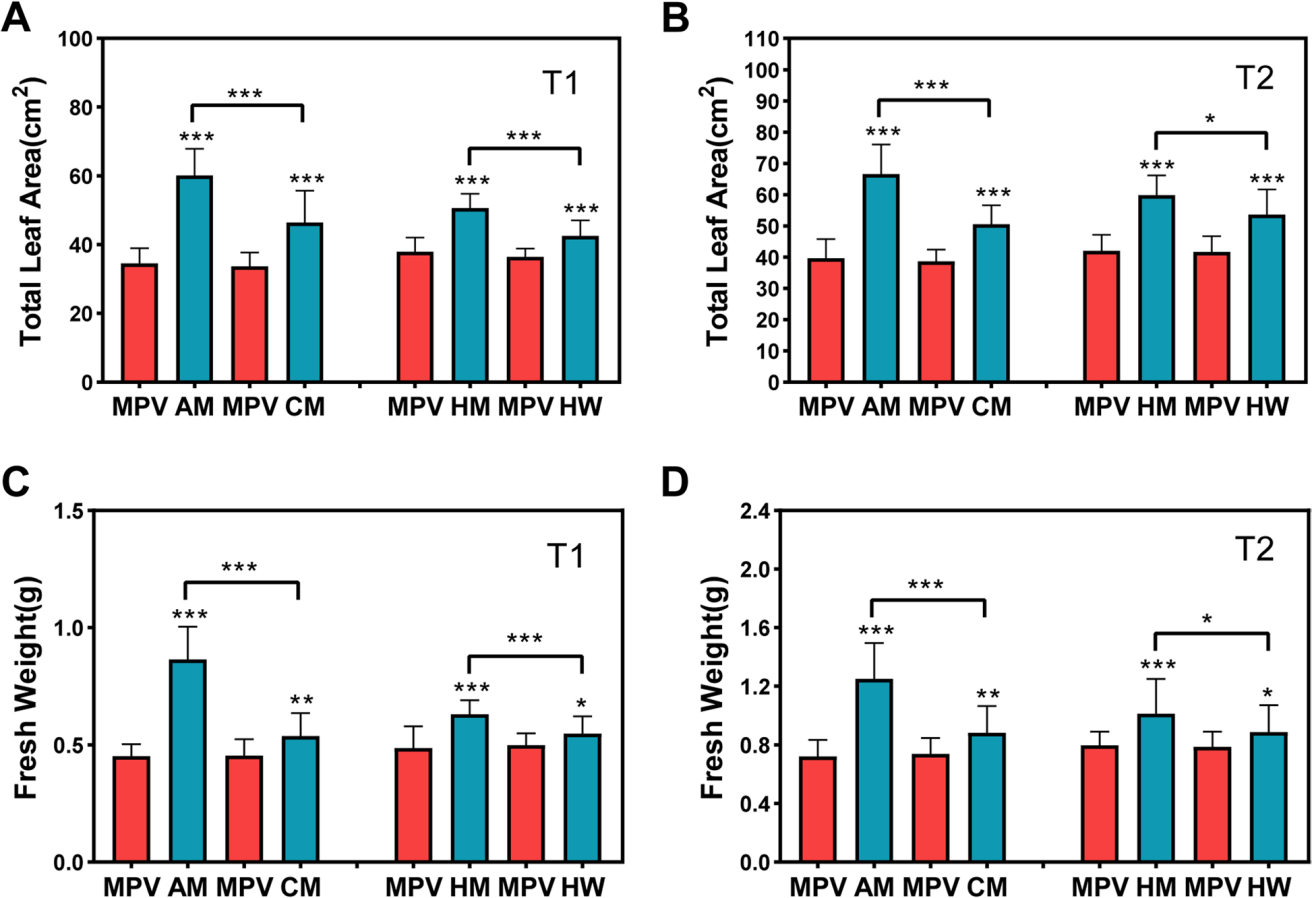
Biomass heterosis was identified in the hybrids in the T1 and T2 periods. **A, B** Total leaf area detected in hybrids with respect to their corresponding MPV at T1 and T2. **C, D** Fresh weight biomass discovered in hybrids and the MPV at T1 and T2. The data are displayed with mean ± standard deviation (SD), derived from the results of three biological replicates. AM, CM, HM, and HW were F_1_ hybrids. MPV: mid-parent value; T1:21 DAS; T2: 24 DAS; *P < 0.05; **P < 0.01; ***P < 0.001

We observed that the total leaf area of all hybrids differed significantly from the corresponding mid-parent value (MPV) at 21 and 24 DAS (Fig. 1A, B; Fig. S1). Furthermore, there were significant differences in the total leaf area of strong hybrid AM relative to CM and the advantageous hybrid HM relative to HW in the two periods (Fig. 1A, B). Interestingly, when we analyzed the fresh weights of the two periods, we observed the similar changes mentioned above (Fig. 1C, D; Fig. S1; Tables S1, S2). The results indicated that AM and HM were vigorous hybrids. The results also showed that the two pairs of hybrids and parents with obvious differences were suitable for the comparative transcriptomic analysis and regulation mechanism study on biomass heterosis at the seedling stage of *B. napus*.

### Identification and analysis of differentially expressed genes (DEGs)

The range of clean reads obtained from all sequencing samples was 33,395,822 to 65,055,276. The clean data of each sample reached 6.52 Gb on average. The range of clean bases for all sequencing samples was 5,009,373,300 to 9,758,291,400. The average Q20 value of our samples was 97.29% and the average GC content was 47.82%. Among the clean reads, the percentage of reads uniquely mapped to the reference genome averaged 79.26% (Table S3). The Pearson correlation coefficient between biological replicates was high, with an average of 98.69% (Fig. S2). This indicates that our transcriptome data are credible.

**Fig. 2:**
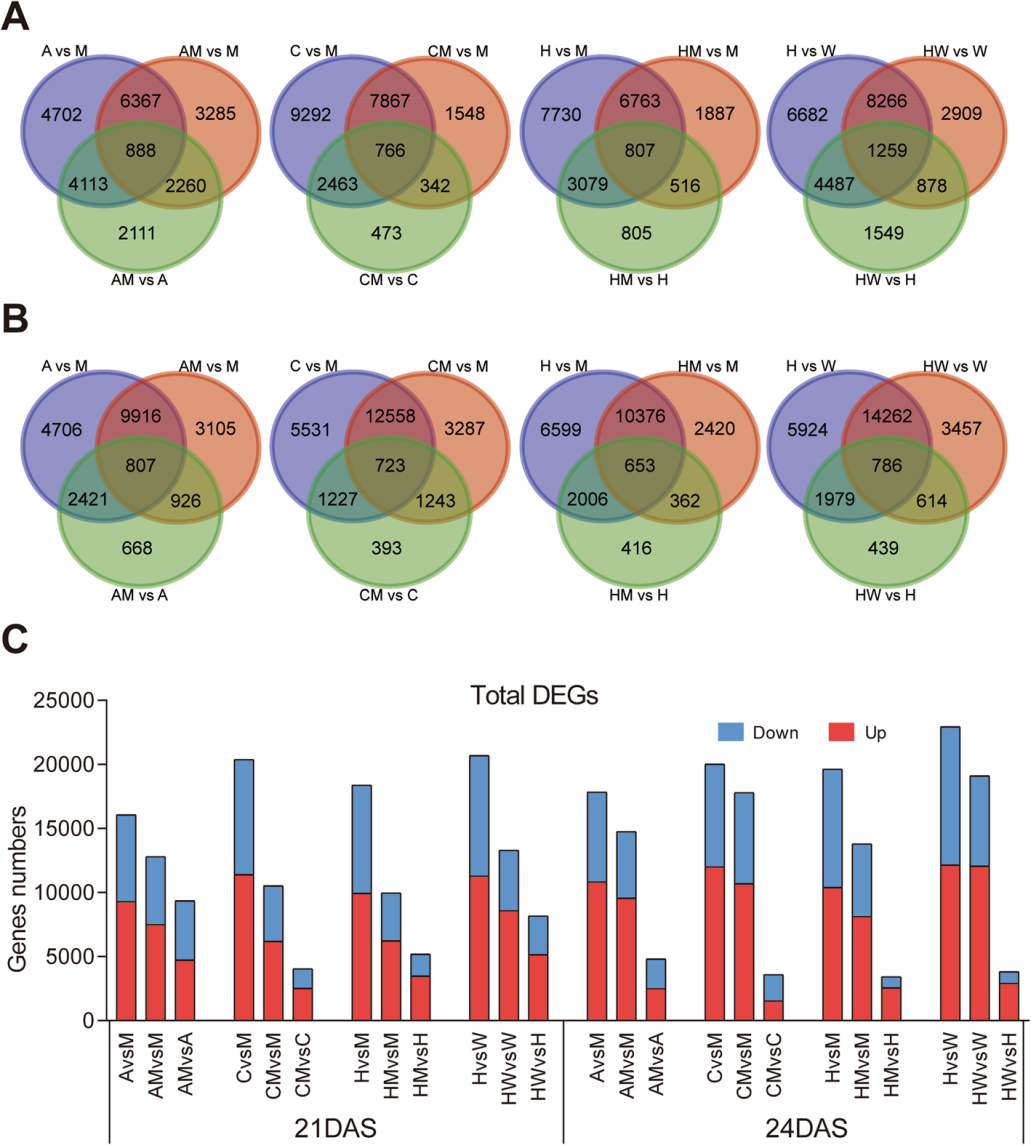
Overview of the DEGs of the four F_1_ hybrids and the parents in two periods. The Venn diagrams **A** and **B** exhibit the allocation of the special and prevalent DEGs of the four hybrids and parents at 21 DAS and 24 DAS, respectively. **C** The overall number of upregulated and downregulated DEGs at 21 DAS and 24 DAS. DEG, differentially expressed gene

Herein, we analyzed the dynamic changes in the transcriptome of two pairs of strong and weak hybrids (AM-CM and HM-HW) and their parents. Venn diagram analysis conducted on the DEGs of 21 DAS and 24 DAS hybrids apropos of their parents (Fig. 2A, B). For example, 21 DAS AMvsA, AMvsM, and AvsM had 2111, 3,285, and 4702 unique DEGs, respectively (Fig. 2A). In addition, 24 DAS AMvsA, AMvsM, and AvsM had 668, 3105, and 4706 unique DEGs, respectively (Fig. 2B). The numbers of upregulated and downregulated genes between F_1_ hybrids and their parents at two periods were further analyzed (Fig. 2C, Tables S4, S5). The results showed that at 21 DAS, the upregulated and downregulated genes in HMvsH, HMvsM, and HvsM were 3492 and 1715, 6247 and 3726, and 9941 and 8438, respectively (Fig. 2C, Tables S4, S5). The total DEGs of HMvsH and HMvsM were 3437 and 13,811 at 24 DAS, respectively (Fig. 2C, Tables S4, S5).

Interestingly, we discovered that the number of upregulated genes in most hybrids was markedly higher than that of downregulated genes (Fig. 2C, Tables S4, S5). The number of DEGs between parents was the highest. The number of DEGs also increased as the plants grew, and more drastic transcriptomic changes were observed at 24 than at 21 DAS. The number of DEGs between all hybrids and the male parents was less than that between the hybrids and female parents (Fig. 2C).

### Analysis of the expression level of hybrids and their parents

**Fig. 3:**
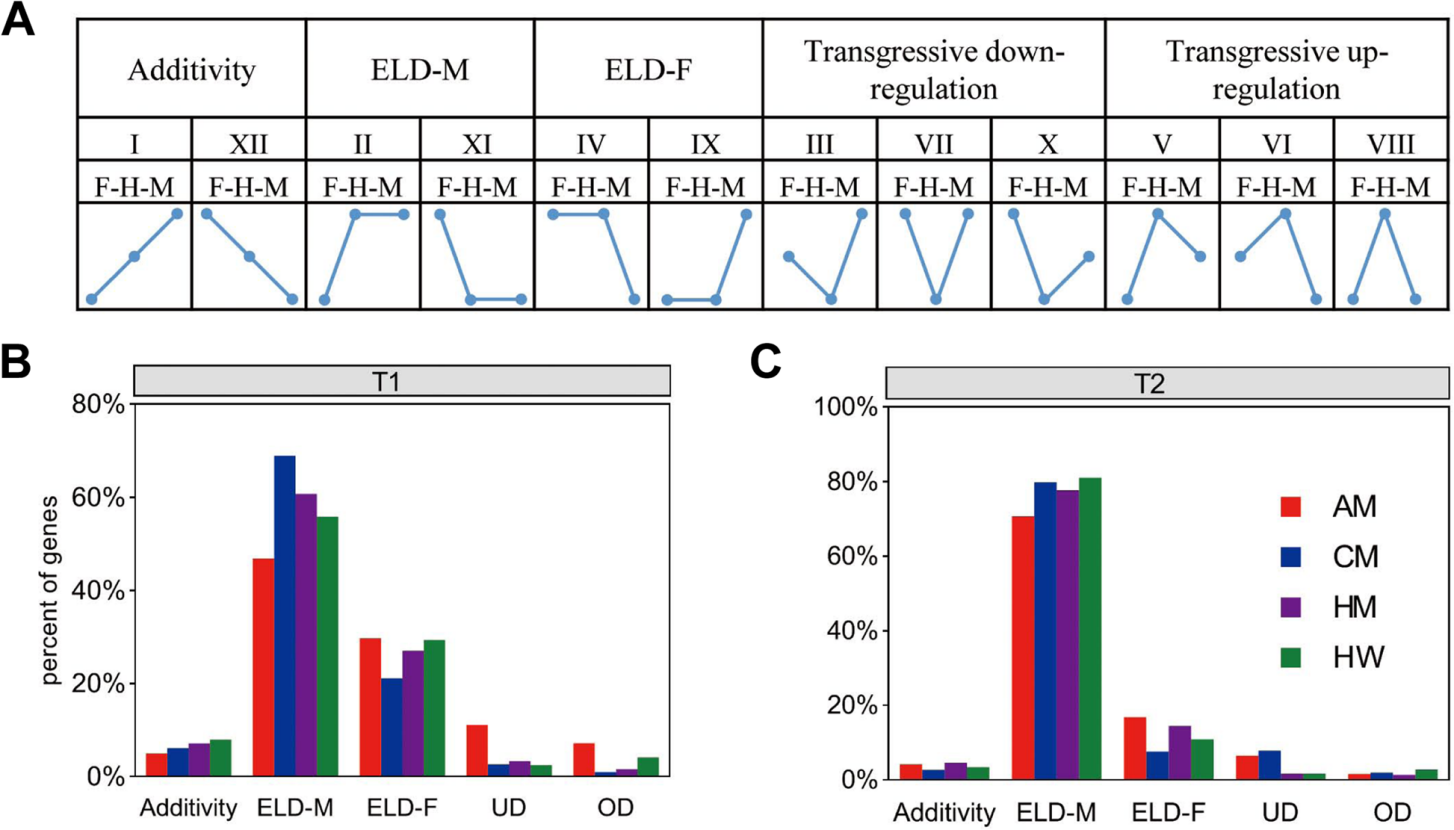
Twelve gene expression types in the canola hybrids contrasted with their parents. **A** Expression types of 12 classes. M: male parent; F: female parent; H: hybrid; ELD-M: The gene expression extent of the F_1_ hybrids was like that of the male parent; ELD-F: the gene expression extent of the F_1_ hybrids was like that of the female parent. **B** and **C** represent the percentage of gene count in the five main categories in the T1 and T2 periods, respectively. T1: 21 DAS; T2: 24 DAS. UD stands for transgressive downregulation; OD stands for transgressive upregulation

To explore the possible changes and directions, we divided the DEGs into 12 possible groups based on previous studies (Fig. 3A) [26, 27]. Many ELD genes were identified at 21 and 24 DAS. Among them, additivity, transgressive downregulation, and transgressive upregulation genes accounted for only a small part of the total (Fig. 3B, C). Most of the gene expression in the hybrids was similar to that of the parents (Fig. 3B, C, Fig. S3, Tables S6, S7). In the two periods examined, ELD male (ELD-M) and ELD female (ELD-F) were classified as having high expression in all hybrids. Among the expressed genes of ELD classification, the parental-ELD genes at 21 and 24 DAS accounted for an average of up to 85% and 90%, respectively (Fig. 3B, C). The genes classified by high parental-ELD in the two periods had a high proportion of 58% and 62%, respectively (Fig. S3). In conclusion, the analysis of expression patterns showed that ELD was ubiquitous. Parental ELD had a strong positive correlation with seedling biomass. In particular, the dominant effect was the main contributor to the leaf growth of hybrids at the seedling stage.

### Gene ontology (GO) enrichment analysis of parental-ELD (P-ELD) gene

To explore the biological function of the P-ELD gene, the four types of genes (II + XI + IV + IX) in each F_1_ hybrid in the two periods were analyzed by GO enrichment analysis (Tables S8, S9). At 21 DAS, the molecular functions of the P-ELD gene of the hybrids were significantly enriched in the structural components of RNA binding and ribosomes when compared to those of the parents (Fig. S4A, Table S8). Through cell component enrichment analysis, we found that they were significantly enriched in chloroplasts and the chloroplast matrix. (Fig. S4B, Table S8). At 24 DAS, the molecular functions of the hybrids relative to the P-ELD gene in both parents were significantly enriched in NAD, NADP binding, and oxidoreductase (Fig. S5A, Table S9). The cell components were significantly enriched in the chloroplast, chloroplast matrix, and photosynthetic membrane (Fig. S5B, Table S9).

We focused on the biological process of the significant enrichment of the P-ELD genes of the four hybrids in the two periods (Tables S8, S9). The Venn diagram showed that the two periods had a common and unique biological pathway (BP) term. The number of significant BP terms in the T2 period (24 DAS) was much greater than that in the T1 period (21 DAS) (Fig. S6A, B). Interestingly, the significantly enriched biological pathways were mainly enriched in photosynthesis (GO:0,015,979), carbon metabolism (GO:0,005,975), plant hormone response (GO:0,009,725), and other pathways at 21 and 24 DAS (Fig. S6C, D).

### Comparative analysis of strong and weak hybrids

**Fig. 4:**
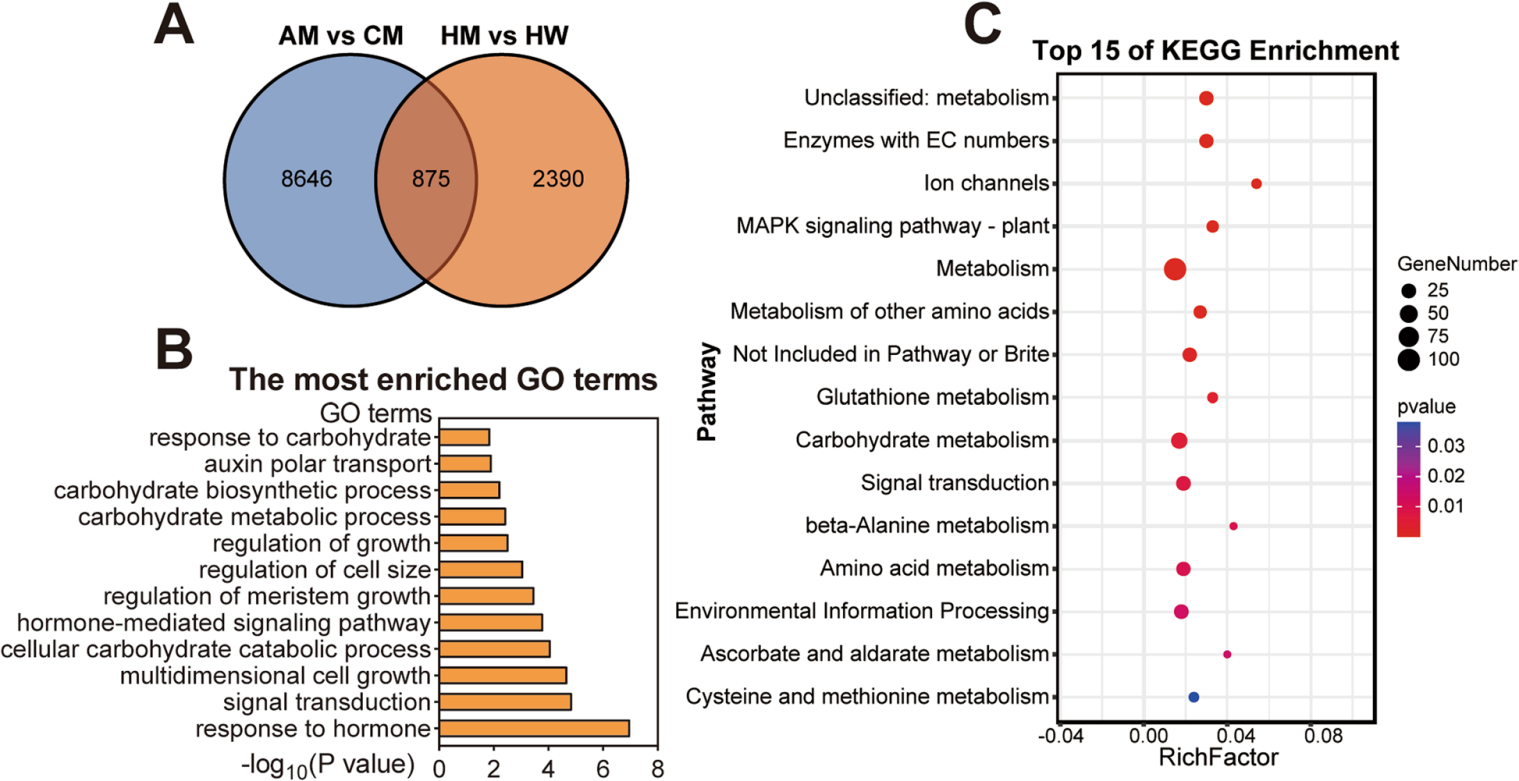
Analysis of differentially expressed genes (DEGs) between strong and weak hybrids at 21 DAS. **A** Venn diagram of the number of unique and shared DEGs between the two groups of strong and weak hybrids at 21 DAS. **B** GO terms with significant enrichment of DEGs shared between the two strong and weak hybrids at 21 DAS. **C** The significantly enriched KEGG terms of DEGs shared between the two strong and weak hybrids at 21 DAS

To explore the changes in strong and weak hybrids, we analyzed the differential expression of two pairs of strong and weak hybrids (AM-CM and HM-HW) in two periods. The two pairs of strong and weak hybrids had 875 DEGs in common at 21 DAS (Fig. 4A, Table S1^0^). The GO enrichment inquiry the common DEGs disclosed that they primarily benefited from biological processes, such as response to hormones, regulation of cell size, and carbohydrate metabolism (Fig. 4B, Table S11). The Kyoto Encyclopedia of Genes and Genomes (KEGG) enrichment analysis of shared DEGs showed they were mainly enriched in the carbohydrate metabolism and signal transduction pathways (Fig. 4C, Table S12).

We performed the same analysis at 24 DAS. A total of 563 DEGs were shared by the two pairs of strong and weak hybrids (Fig. S7A, Table S13). The GO enrichment analysis of these shared DEGs demonstrated that they were largely enriched in the biological processes of hormone metabolism, circadian rhythm, and carbohydrate metabolism (Fig. S7B, Table S14). The KEGG enrichment analysis performed on the shared DEGs showed that they were mainly enriched in pathways such as carbohydrate metabolism and starch and sucrose metabolism (Fig. S7C, Table S15). These outcomes suggest that the contrasting expression of these pathways can lead to differences between strong and weak hybrids.

### Analysis of plant hormone transduction between hybrids and parents

**Fig. 5:**
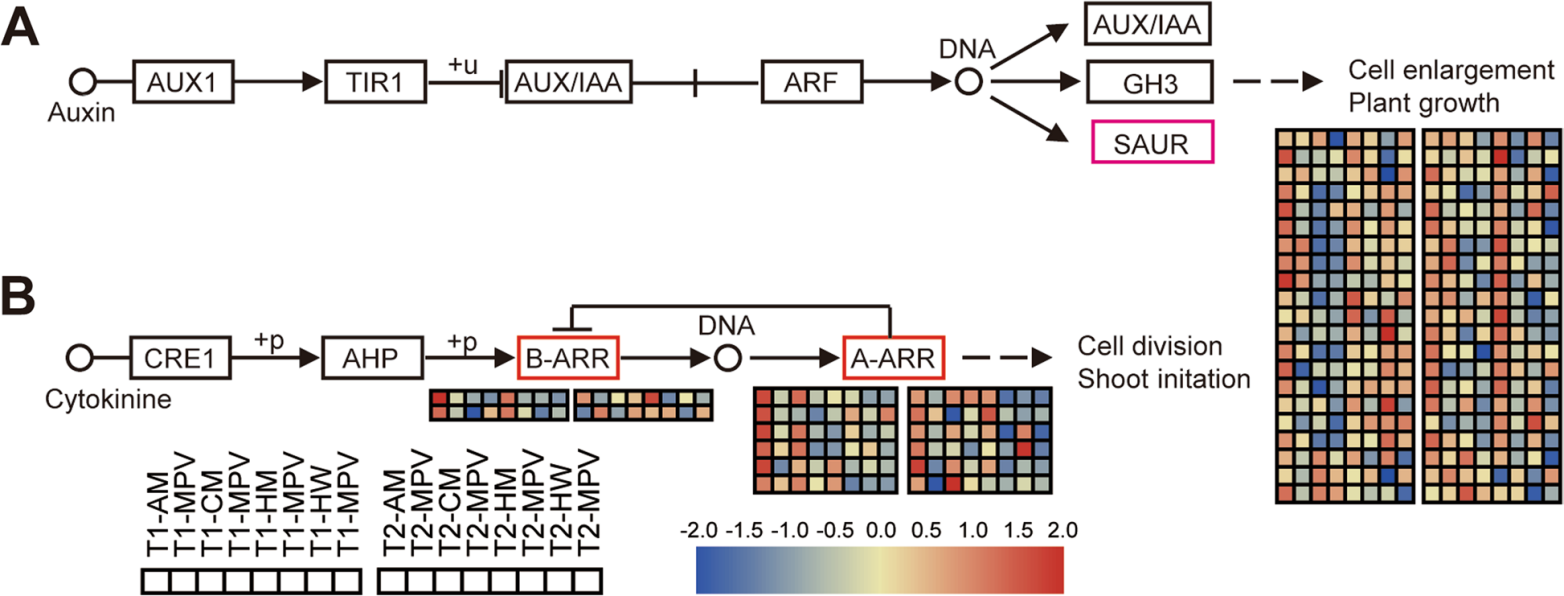
Heat map of DEGs among hybrids and parents in the plant hormone signal transduction pathway. DEGs with significant differences are marked in red. Panels **A** and **B** display heat maps of the expression changes of DEGs in the auxin and cytokinin signal transduction pathways, respectively. T1: 21 DAS; T2: 24 DAS; MPV: mid-parent value; SAUR: small auxin-upregulated RNA; B-ARR: type B-*Arabidopsis* response regulator; A-ARR: type A-*Arabidopsis* response regulator

According to the enriched biological process, the plant hormone signal transduction pathway was significantly enriched (Fig. S6C, D). We focused on genes related to the auxin and cytokinin transduction pathways (Tables S8, S9). The regulatory pathways are shown in Fig. 5a and b. Significant differences in small auxin upregulated RNA (SAUR) were observed in the auxin signaling pathway, including SAUR4, SAUR14, SAUR15, SAUR19, SAUR20, SAUR21, SAUR23, SAUR33, and SAUR51 (Fig. 5A). Interestingly, based on our analysis, up to 10 SAUR21 genes (*BnaA03g06810D, BnaA10g16790D, BnaA10g16800D, BnaC02g07800D, BnaC03g08730D, BnaC03g08740D, BnaC03g08750D, BnaC03g08780D, BnaC09g39830D,* and *BnaC09g52070D*) in the pathway were significantly expressed in the hybrids, and they were expressed in high amounts relative to the MPV.

We compared the alterations in the gene expression of the four hybrids (AM, CM, HM, and HW) relative to MPV in two periods (T1 and T2) and observed significant differential expression of the genes of B-Arabidopsis Response Regulators (ARR) and A-ARR (Fig. 5B). Notably, the B-ARR gene was predominantly expressed in AM and HM hybrids relative to MPV; the same gene was more highly expressed in AM and HM hybrids than in CM and HW hybrids. Similar changes were observed in A-ARR genes. These results indicate that genes in the signal transduction pathway of auxin and cytokinin may change leaf expansion and eventually evoke changes in hybrid rape biomass vigor.

### Changes in cell size-related gene expression and leaf phenotypes in hybrids

**Fig. 6:**
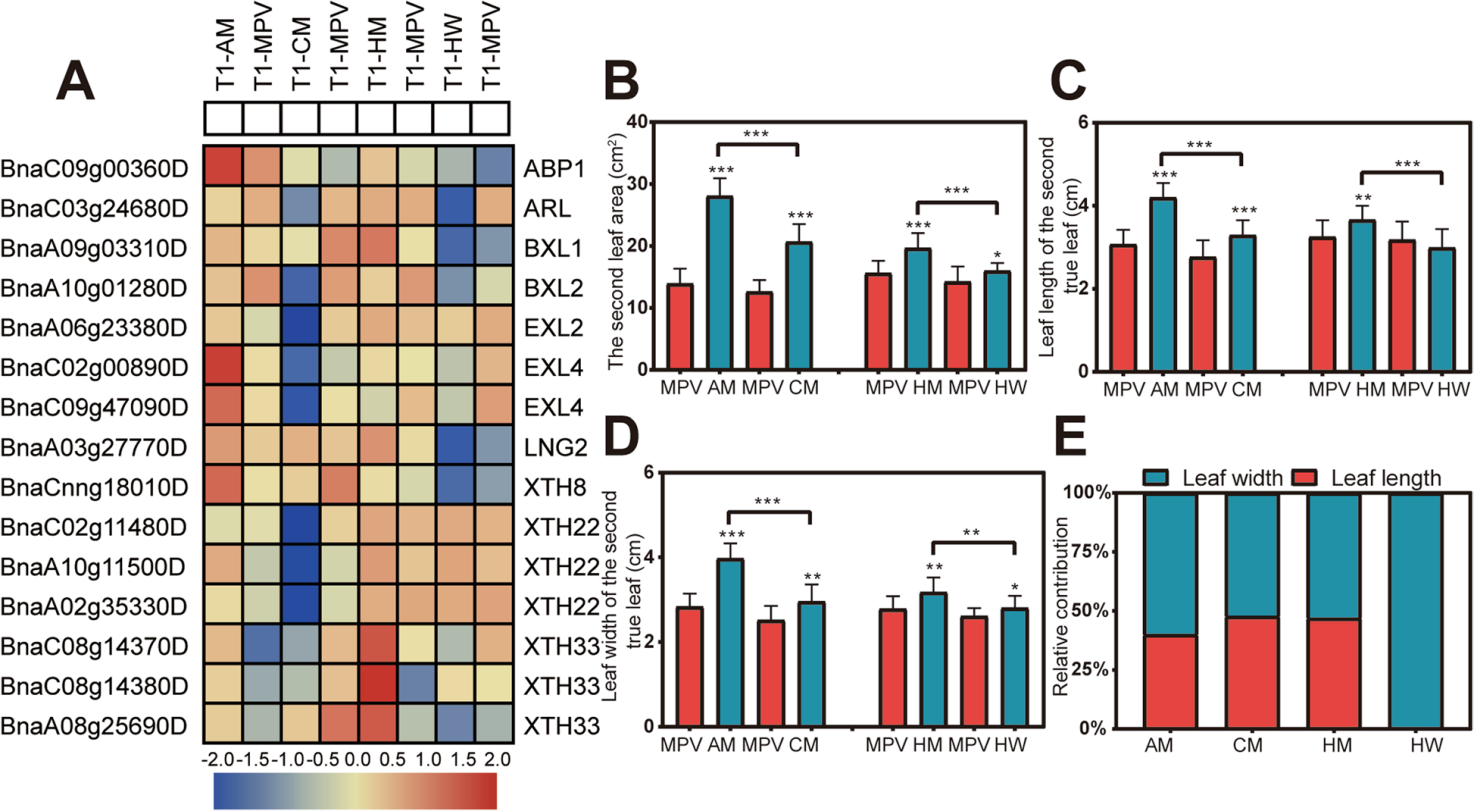
Related phenotypic changes in the second true leaf of hybrids and their parents at 21DAS. **A** Heat map of genes that promoted cell size in F_1_ hybrids relative to their parents at 21 DAS. The second true leaf area **B**, leaf length **C**, and leaf width **D** of all the F_1_ hybrids and their MPV at 21 DAS. **E** Histogram showing the percentage of contribution of leaf length and leaf breadth to the second true leaf area in four hybrids at 21 DAS. The data are expressed as mean ± SD, derived from the results of three biological replicates. AM, CM, HM, and HW are F_1_ hybrids; MPV, mid-parent value; T1: 21 DAS; *P < 0.05; **P < 0.01; ***P < 0.001

Many of the DEGs of hybrids promoted an increase in the cell size (Fig. 6A). Compared with those in the CM and HW hybrids, the genes that promoted cell size in the strong hybrids AM and HM were mainly upregulated and more highly expressed (Fig. 6A). Hybrids with heterosis at the seedling stage were likely to have either or both increased leaf length and leaf width. Consistent with the results of biomass, the AM and HM hybrids had significant heterosis in the second true leaf area relative to the MPV and had significant differences between AMvsCM and HMvsHW (Fig. 6B). The AM, CM, and HM hybrids showed an increase in leaf length and width correlated with the MPV (Fig. 6C, D). We found that the contribution of leaf width to the leaf area of ​​the enlarged second true leaf was greater than that of the leaf length (Fig. 6E). By performing the same analysis as above on the third true leaf at 24 DAS (Fig. S8, S9), we discovered that genes related to leaf cell size, such as BXL1 (*BnaA02g30740D* and *BnaC02g39030D*), LNG2 (*BnaC03g32810D*), and PIF4 (*BnaA03g19970D, BnaC04g48630D, BnaA04g24760D,* and *BnaC01g40470D*) were highly expressed in strong dominant hybrids (AM and HM) and significantly different in the CM and HW hybrids (Fig. S8). Similar results were obtained regarding the phenotypic data of the third true leaf area, leaf length, and breadth (Fig. S9A, B, C). Except for that to the HW hybrid, the contribution of leaf width to the leaf area of other hybrids was greater than that of leaf length (Fig. S9D).

### Comparison among genes in the photosynthetic pathways of hybrids

**Fig. 7:**
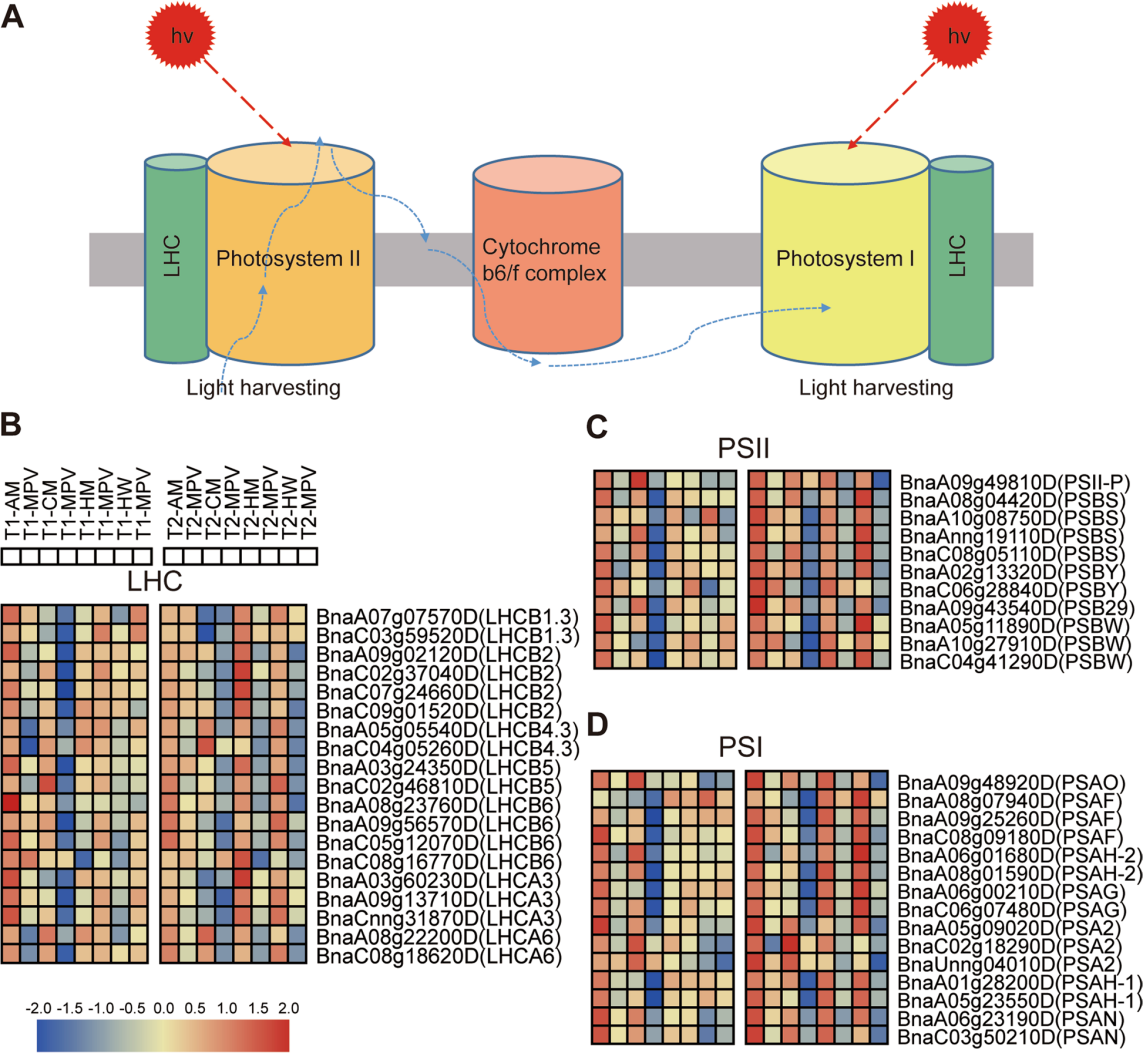
Photosynthetic expression of hybrids relative to their parents changed during the T1 and T2 periods. **A** A schematic diagram of the expression pattern of photosynthesis. **B-D** Heat maps of the key genes in the photosynthetic enrichment pathways related to LHC, PSII, and PSI, respectively, in the two periods. Red and blue represent upregulation and downregulation, respectively. The figure shows the expression changes in the hybrids (AM, CM, HM, and HW) relative to the corresponding MPV genes in the two periods. T1: 21 DAS; T2: 24 DAS; MPV, mid-parent value; LHC, light-harvesting complex; PSII, photosystem II; PSI, photosystem I

According to previous reports, photosynthesis is very important for plant heterosis [7, 12]. The significant enrichment process of many P-ELD genes was related to photosynthesis (Fig. 7A), and several DEGs constituted photosystem I, photosystem II, and light-harvesting complex (LHC) (Fig. 7B, C, D; Tables S8, S9). We analyzed the changes in DEGs related to the photosynthesis of hybrids and their corresponding MPVs in the T1 and T2 periods (Fig. 7B, C, D). For LHC, we observed that the genes of *BnaA09g02120D* (LHCB2), *BnaC09g01520D* (LHCB2), *BnaA05g05540D* (LHCB4.3), *BnaC04g05260D* (LHCB4.3), and *BnaA08g22200D* (LHCA6) showed upregulated expression relative to the MPV at 21 and 24 DAS. AM had higher expression levels of these genes than CM, and HM had higher expression levels of these genes than HW (Fig. 7B). Moreover, the PSII and PSI genes showed similar expression trends (Fig. 7C, D). The results showed that the upregulated expression of genes associated with photosynthesis contributes to the production of biomass heterosis.

### Correlation network analysis of leaf development in hybrids and parents at the seedling stage

Results suggested that the DEGs shared by all hybrids and parents are closely related to the biomass phenotype in the two periods. Consequently, weighted gene co-expression network analysis (WGCNA) was executed for related genes in all samples to identify the key genes and underlying mechanism highly related to the biomass phenotype. Ultimately, 17 modules were identified, as shown in the dendrogram (Fig. S10). The eigengenes of the 17 modules were associated with different samples (Fig. S11).

This study focused specifically on the module closely related to leaf development in the strong heterosis hybrid. Therefore, we associated modules with different samples. We discovered that the yellow module was markedly correlated with biomass (Fig. S11). The yellow module contained 487 genes and highly expressed transcripts in the vigorous hybrid (AM) (Fig. S12, Table S16). KEGG enrichment analysis was conducted on the genes of this module, which were primarily enriched in plant hormone signal transduction and various metabolic processes (Fig. S13, Table S17).

To further analyze the yellow module, we identified 53 hub genes (membership degree value [KME] > 0.90, *P* < 10^–6^) (Table S18). GO enrichment analysis indicated that these hub genes were overexpressed in functional categories related to the regulation of plant growth and development, such as hormone-mediated signal transduction, the response to auxin, and regulation of the hormone level (Fig. S14, Table S19). These results support the role of the hub genes in early leaf growth and development.

### qRT-PCR verification of the transcriptome

**Fig. 8:**
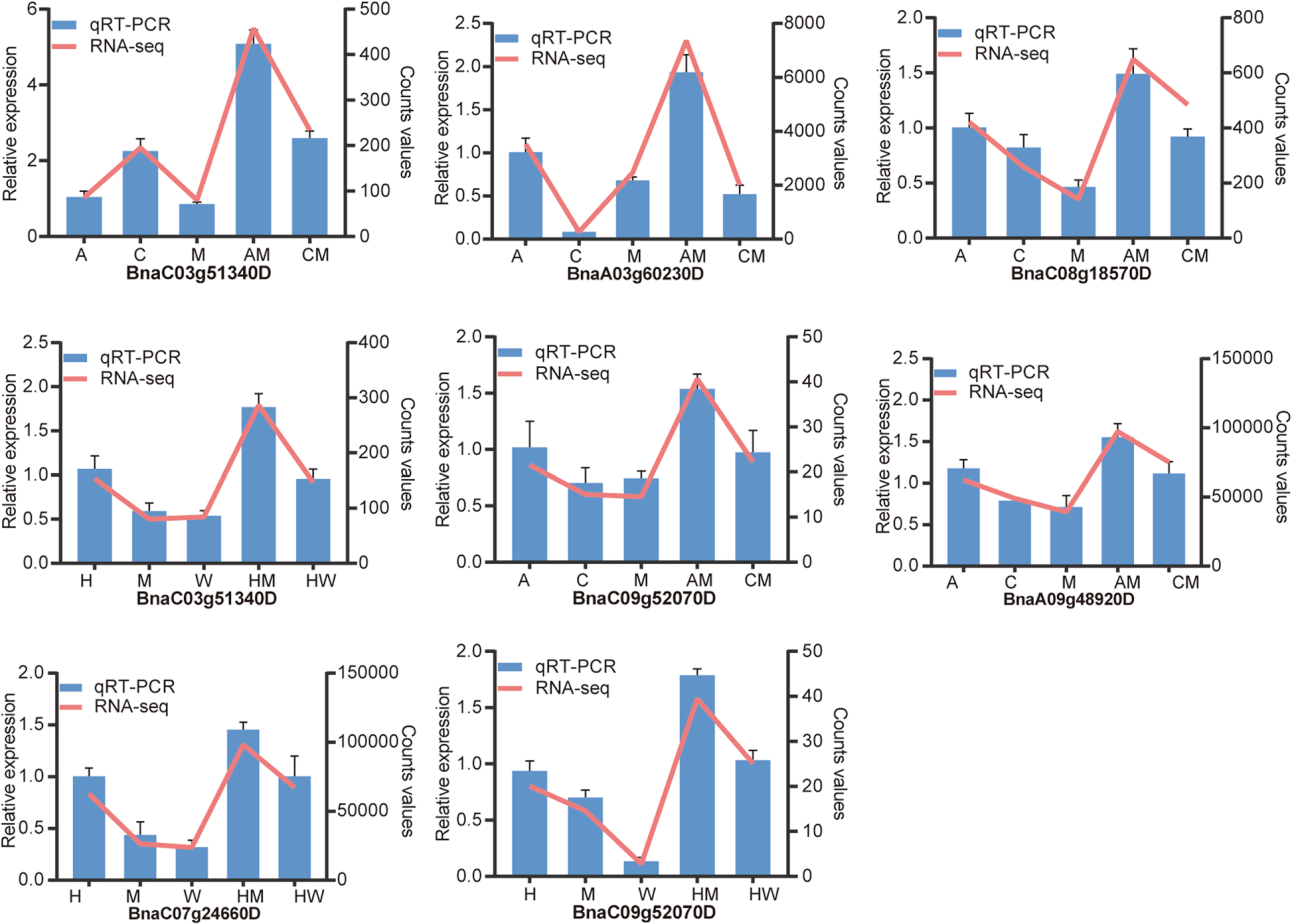
qRT-PCR validates the expression of eight genes in photosynthetic and plant hormone signal transduction pathways. The blue histogram shows the relative expression levels (mean ± SD) of genes obtained through quantitative verification. The red line graph illustrates the gene expression level value obtained by the transcriptome. Male: A, C, H; Female: M, W; Hybrid F_1_: AM, CM, HM, HW. The first four genes were expressed at 21 DAS, whereas the other genes were expressed at 24 DAS

We randomly selected nine candidate genes in the transcriptomic data at 21 and 24 DAS to perform qRT-PCR experiments to certify the reliability of the transcriptomic outcomes. Nine genes, including *BnaC07g24660D, BnaA09g49810D, BnaC06g28840D,* and *BnaA09g48920D*, were used for quantitative analysis. Detailed information on the primers used is shown in Table S20. The quantitative results were highly consistent with the trends of the transcriptomic data, further confirming the accuracy of transcriptomic outcomes (Fig. 8 and Fig. S15).

## Discussion

Heterosis is a widely known event wherein hybrids display vigor greater than that of their parents [11, 28]. The vegetative growth of plant seedlings is critical to the increase in yield at a later time, and the advantage of the seedling stage is to eliminate environmental interference and better reflect the mechanism underlying heterosis. In this study, we used comparative transcriptomic analysis of the leaves of two pairs of hybrids and their parents (AM-CM and HM-HW) at two stages and explored the molecular mechanism underlying biomass heterosis at the seedling stage.

**Fig. 9:**
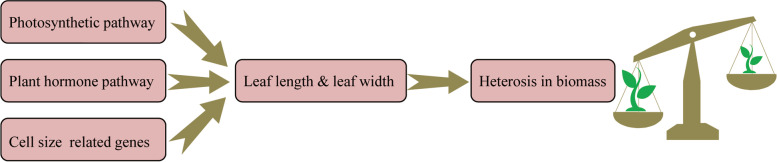
Model explaining the heterosis of seedling biomass in canola hybrids. In the early stages of seedling growth, the photosynthetic and plant hormone signal transduction pathways along with the high parental dominance expression of key genes correlated with cell size, ultimately lead to the differential expression of hybrids in terms of leaf length and leaf width relative to their parents. Subsequently, we observe heterosis in the biomass. Moreover, the expression levels of these vital genes are different in strong and weak hybrids, which also explains the phenotypic heterogeneity of strong and weak hybrids

Interestingly, we measured the leaf length and width of the second and third true leaves at the same time point at 20–30 DAS and found that the days with the fastest daily growth rate of the second and third true leaves were 21 DAS and 24 DAS, respectively (Fig. S16). Therefore, we analyzed the transcriptomic data of the second and third true leaves in two sampling periods from four hybrids (AM, CM, HM, and HW) and their parents (A, C, H, M, and W). Herein, we propose a model in which we observe the differential expression of genes in the photosynthetic pathway and the plant hormone signal transduction pathway between hybrids and parents. The changes in the cell size-related genes led to changes in leaf length and leaf width and ultimately affected the differences in biomass heterosis between strong and weak hybrids, as well as between the hybrids and their parents (Fig. 9).

### Possible roles of hormone transduction pathway genes in seedling heterosis

In the present study, two strong rape hybrid combinations (AM and HM) in the auxin and cytokinin signal transduction pathways (especially in the former) exhibited many genes with upregulated expression relative to their parents. Recent studies have provided evidence for a correlation between auxin and cytokinin and heterosis. The high expression of auxin-related genes has played a positive role in promoting heterosis in crops such as *Arabidopsis* [8], maize [29], and cotton [30]. These reports further support our conclusions. We detected the differential expression of the *SAUR* gene in the auxin signal transduction pathway. Consistent with our results, Huang et al. who analyzed the size of *Euryale ferox* Salisb hybrid seeds, revealed that the *SAUR* gene can affect the distribution of auxin and play a potential regulatory role in hybrid seed size [31].

Cytokinins play a crucial role in regulating plant growth and development. They can promote the proliferation and expansion of leaf cell development and coordinate with auxins to regulate plant growth [32]. In our study, two genes related to the response regulation of *Arabidopsis* were upregulated in strongly dominant hybrids. These genes are thought to be related to an increase in biomass. However, the factors that upregulate auxin and cytokinin signal transduction pathway genes in hybrids remain unclear. The differential expression of different genes in plant IAA and cytokinin signal transduction pathways may lead to different levels of hybrid seedling vigor. This indicates the high expression of genes related to auxin and cytokinin signaling pathways in hybrids contributes to the occurrence of heterosis in canola at the seedling stage.

### Role of cell size-related genes in plant growth and development

Reports that focus on the relationship between cell size-related genes and heterosis are of particular interest. In poplars, the advantage of leaf size in triploid hybrids is positively correlated with an increase in the expression of genes regulating cell size [33]. In *Arabidopsis*, changes related to genes of leaf size during hybrid rosette development have been observed [34] as well as the upregulated expression of xyloglucan endotransglucosylase/hydrolase-related genes in the three hybrid systems [35]. However, the factors that drive the upregulation of cell size-related genes in hybrids remain unclear. In our seedling transcriptomic data, genes that promote cell size were highly expressed in rape hybrids relative to their parents. In addition, we provided further physiological evidence that hybrids have more advantages in terms of leaf area than their parents. The leaf length and leaf width of the hybrids and parents were further measured. The difference between the strong and weak hybrids of the same male parent and different female parents may be caused by maternal effects in the early stages of development. This effect on heterosis may indicate there are various cytoplasmic interactions between different parents.

### Photosynthesis plays a crucial role in early biomass hybrid vigor

Photosynthesis is an essential basic approach, which supplies plants with energy and helps balance the energy distribution [36]. In Brassicaceae hybrids, the early activation of related genes in the photosynthetic pathway is universal. In *Arabidopsis*, photosynthesis significantly promotes an increase in the heterosis of hybrid biomass [7, 21, 37]. In Chinese cabbage hybrid seedlings, high parental expression of related genes in hybrids during photosynthesis, such as in photosystem I and photosystem II, has been detected [7, 38]. Our results show that in rape hybrids, the photosynthesis genes were significantly expressed in strong dominant biomass hybrids at 21 and 24 DAS relative to their parents. In addition, we compared the gene expression changes in the photosynthetic pathway between strong and weak hybrids. Based on our results, it is not yet possible to explain whether the contribution of the female or male parent to the hybrid is superior, and further research is required. Subsequent methylation sequencing can be performed on hybrids and parents to explore whether the promoter region of the core gene of interest is regulated by methylation or demethylation. This affects the differential expression of core genes, which may lead to the dominant phenotype of the hybrid.

## Conclusions

We analyzed the transcriptomic data of different strong and weak hybrids and their parents at multiple time points to illustrate the important role of dominant genes with high parental expression levels, which are mainly involved in the formation of biomass heterosis in *B. napus* seedlings through plant hormone signal transduction and the photosynthetic pathway. However, our results do not negate other possible situations that affect seedling heterosis. Future studies should explore the mechanisms through which the genes of related pathways promote heterosis production. This study provides novel insights into biomass heterosis during the vegetative stage and may help provide theoretical support for the cross-breeding of canola.

## Methods

### Plant samples and growth situations

The seeds used in this experiment were provided by the rape Laboratory of Huazhong Agricultural University and created by Dr. Zhao [39, _1_]. Through phenotypic analysis of 30 DAS biomass heterosis [_1_], we screened strong and weak hybrid combinations with the same parents. Finally, two pairs of strong and weak hybrids (AMvsCM and HMvsHW) and their five inbred parents (A, C, H, M, and W) were used to study biomass heterosis. M and W were female parents; A, C, and H were male parents; and AM, CM, HM, and HW were F^40^ hybrids. Among them, the female parent was sterile and male parent was fertile. All materials germinated in a greenhouse in ordered surroundings (temperature: 21–23 °C, humidity: 30–60%, 16 h light/8 h darkness) under the support of sterile gauze. Seven days later, seedlings with consistent growth were selected for hydroponic growth [41]. During this period, the nutrient solution was changed every seven days. After two weeks, the second and third true leaves of all experimental materials were collected at 21 and 24 DAS, respectively. After the samples were collected, they were instantly placed in liquid nitrogen and transferred to -80 °C for storage. Three biological replicates were obtained for each sampling, and each biological replicate contained at least 8–10 samples of the same material. F^42^ hybrids and the parents used in the experiment were placed in the same pot.

### Measurement and analysis of phenotypic traits

The fresh weight, total leaf area, and dry weight of the parents and hybrids were measured at 21 and 24 DAS. Three biological replicates were used, each containing at least 8–10 seedlings. At 21 and 24 DAS, the leaf length, leaf width, and leaf area of the second (third) true leaves of the parents and hybrids were measured. The dry weight was measured by initially measuring the fresh weight; next, the samples were placed in a craft paper bag, heated at 105 °C for 30 min, and then dried at 80 °C to an invariant weight. The fresh and dry weights were measured using an electronic balance. Leaf area was evaluated using ImageJ (http://rsb.info.nih.gov/ij/).

### RNA sequencing

Total RNA was obtained from the materials using the TRIzol method. After total RNA collection and DNase I therapy, RNA degradation and contamination were detected on a 1% agarose gel solution. NanoDrop (Thermo Fisher Scientific, Inc., Waltham, MA, USA) and Agilent 2100 (Agilent Technologies, Santa Clara, CA, USA) were used to verify the purity and completeness of the RNA. Library building and RNA-seq were conducted by the BGI Biotechnology Company (Wuhan, China) under the manufacturer’s guidance. All libraries were sequenced to achieve 150 nucleotide paired-end reads on a HiSeq 4000 platform (Illumina, San Diego, CA, USA).

### Analysis of DEGs

Raw data from the Illumina HiSeq 4000 sequencer was collated to obtain clean data by eliminating reads comprising adapters (N > 10%) or reads with low-quality nucleotides (> 50%) using fastp v0.19.4 software [43]. The clean data were aligned to the “Darmor-bzh” reference genomes [23] using the HISAT2 v2.1.0 software [44]. Finally, the uniquely mapped reads were obtained. Subsequently, the reads mapped to genes or exons were applied to count the gene expression level using FeatureCounts v1.5.0 software and illustrated with transcripts per kilobase of exon model per million mapped reads (TPM) [45]. Genes with > 30 counts from the sum of the three biological replicates were considered expressed genes. The DESeq2 R package was used to analyze DEGs [46]. The DEGs were selected with a false discovery rate (FDR) < 0.05 and |log2(fold change)|> 1. We also calculated Pearson correlation coefficients between pairwise samples with the TPM of all expressed genes.

### Weighted gene co-expression network analysis

WGCNA was performed on differentially expressed genes to explore co-expression modules that were highly correlated with biomass. Using the correlation network of the WGCNA R package, correlation analysis between highly correlated DEGs and samples was carried out to identify modules with high specific expression [47]. The first principal component of the expression profile of each module was calculated and expressed based on the module eigengenes, which were used to estimate the association between the module and the biomass phenotype of different samples. Moreover, the KME of each gene in the module was used to mine the hub gene.

### GO and KEGG analysis

The complete GO annotation information of *B. napus* was annotated according to the description by Wu et al. [48]. GO and KEGG [49] enrichment investigation of DEGs was executed by the Tbtools v1.098689 software [50]. The functional classes with a corrected *p*-value < 0.05 were subsequently analyzed.

### Quantitative analysis using qRT-PCR

To assess the reliability of the transcriptomic data, we randomly selected nine genes in two periods for quantification. PerlPrimer software was deployed by constructing gene-specific quantitative primers. The original sequencing sample was used for quantitative analysis. First-strand cDNA synthesis was performed using DNase I management reagent (DP441; Tiangen, Beijing, China) and a first-strand cDNA synthesis kit (K1622; Thermo Fisher Scientific, Inc.). qRT-PCR was performed using SYBR Green Real-Time PCR Master Mix (Toyobo, Osaka, Japan) and a CFX96 real-time system (Bio-Rad, Hercules, CA, USA). UBC10 was used as a housekeeping gene. The 2^−ΔΔCT^ method was used to analyze the data [51]. All reactions were performed for three biological replicates, and every biological replicate contained three technical replicates.

### Statistical analysis

MPH and HPH were estimated using the following formula: MPH = (F1-MP)/MP × 100%, HPH = (F1-HP)/HP × 100%, where MP is defined as the average performance of two parents, and HP is defined as the performance of the best parent. GraphPad Prism 8 (GraphPad Software, Inc.) was used to analyze the significant differences between hybrid and mid-parent values and strong and weak hybrids using unpaired *t*-tests.

## Supplementary Information

**Additional file 1:** **Figure S1.** Photos showing the seedling phenotypes of the parents and hybrids in the T1 and T2 periods.T1: 21DAS; T2: 24 DAS; Male parent: A, C, H; Female parent: M, W; Hybrid F_1_:AM, CM, HM, HW. **Figure S2.** Pearson correlation heat map between all RNA sequencing samples. The red color indicates a higher correlation between samples. -1, -2, and -3 represent three biological replicates at each time point. T1: 21 DAS; T2: 24DAS; -l: leaf. **Figure S3.** Number of the expression level of dominant(ELD) genes in hybrid canola seedlings in each of the 12 DEG types in the T1 and T2 periods. Genes with an expression degree in the F_1_ hybrids analogous to that of the female parent are defined as ELD-F; genes with an expression degree in the F_1 _hybrids analogous to that of the male parent are defined as ELD-M. T1: 21 DAS;T2: 24 DAS. **Figure S4.** Venn diagram analysis of the significant molecular function (MF) and cellular composition (CC) of the parental-ELD gene at 21 DAS. **A** and **B** display Venn diagrams of MF and CC, respectively. MF: molecular function; CC: cell component **Figure S5.** Venn diagram analysis of the significant molecular function (MF) andcellular composition (CC) of the parental-ELD gene at 24 DAS. **A** and **B** show Venn diagrams of MF and CC, respectively. MF: molecular function; CC: cell component. **Figure S6.** Remarkable biological processes of the parental-ELD gene at T1 and T2 in different hybrids.**A** and **B** exhibit Venn diagrams of the parental-ELD with significantly enriched BP in theT1 and T2 periods, respectively. **C** and **D** show heat maps of the BP terms where the parental-ELD gene is significantly enriched in different hybrids at 21 DAS and 24 DAS, respectively. T1: 21 DAS; T2: 24 DAS. **Figure S7.** Analysis of differentially expressed genes between strong and weak hybrids at 24 DAS.**A** Venn diagram of the number of unique and shared DEGs between the two groups of strong and weak hybrids at 24 DAS. **B** GO terms with significant enrichment of DEGs shared between the two strong and weak hybrids at 24 DAS. **C** The significantly enriched KEGG terms of DEGs shared between the two strong and weak hybrids at 24 DAS. **Figure S8**. Heat map of genes that promoted cell size in F_1_ hybrids relative to their parents at 24 DAS. T2: 24 DAS. **Figure S9. **Phenotypic changes of the leaf area of the third true leaf of all hybrids relative to their parents at 24 DAS. The third true leaf area **A**, leaf length **B**, and leaf width **C** of all F_1_ hybrids and their MPV at 24 DAS. **D**,The histogram shows the percentage of the contribution of leaf length and leaf width to the third true leaf area in four hybrids at 24 DAS. The data are expressed as mean ± SD, derived from the results of three biological replicates. AM, CM, HM, and HW are F_1_ hybrids; MPV: mid-parent value; *P<0.05; **P<0.01; ***P<0.001. **Figure S10. **Hierarchical clustering tree of 17 modules obtained by WGCNA.** Figure S11. **Heatmap of the correlation analysis of modular traits. Each row represents a module and each column represents a different sample. The correlation coefficients and P-values are shown in the figure. Red and green represent positive and negative correlations, respectively.** Figure S12. **Heatmap showing the eigengene expression profile of the yellow module in different samples. **Figure S13.** **S**ignificantly enriched KEGG terms in yellow modules. **Figure S14.** Circle chart of GO terms showing significant enrichment of hub genes in the yellow module. The first circle is the enriched classification, and the outer circle is the coordinate ruler of the number of genes. The second circle is the number of the category in the background gene. The third circle is the number enriched in the category. The fourth circle is the value of the rich factor for each category. **Figure S15. **qRT-PCR validation of the expression degrees of 10 genes in the photosynthetic and plant hormone signal transduction pathways. The blue histogram shows the relative expression levels (mean ± SD) of genes obtained through quantitative verification. The red line graph depicts the gene expression level value obtained by the transcriptome. Male: A, C, H; Female: M, W; F_1 _hybrid: AM, CM, HM, HW. The first four genes were expressed at 21 DAS, whereas the other genes were expressed at 24 DAS. **Figure S16.** Determination of the leaf sampling period. Figures **A** and **B** represent the line graphs of the measured daily growth rate of the leaf length and breadth of the second and third true leaves, respectively, at 20–30 DAS. 

**Additional file 2:** **Table S1.** Phenotypic data of T1 are expressed as mean ± SD, T1 refers to 21 DAS. The data represents the results of three biological repetitions. **Table S2.** Phenotypic data of T2 are expressed as mean ± SD, T2 refers to 24 DAS. The data represents the results of three biological repetitions. **Table S3.** Quality evaluation of RNA-seq reads and mapped to reference genome. **Table S4.** Information on upregulated and downregulated DEGs of 21 DAS hybrid F1 (AM, CM, HM, HW) and their parents (A, C, H, M, W). **Table S5.** Information on upregulated and downregulated DEGs of 24 DAS hybrid F1 (AM, CM, HM, HW) and their parents (A, C, H, M, W). **Table S6.** Specific gene information for the classification of expression patterns of 12 types of ELD genes at 21 DAS. **Table S7.** Specific gene information for the classification of expression patterns of 12 types of ELD genes at 24 DAS. **Table S8.** Results of significant GO enrichment of F1 relative to the parent of the parental-ELD gene at 21 DAS. **Table S9.** Results of significant GO enrichment of F1 relative to the parent of the parental-ELD gene at 24 DAS. **Table S10.** Information on DEGs of strong hybrids and weak hybrids at 21 DAS. **Table S11.** The results of the significantly enriched GO terms of the DEGs shared by the two pairs of strong and weak hybrids at 21 DAS. **Table S12.** The results of the significantly enriched KEGG pathways of the DEGs shared by the two pairs of strong and weak hybrids at 21 DAS. **Table S13.** Information on DEGs of strong hybrids and weak hybrids at 24 DAS. **Table S14.** The results of the significantly enriched GO terms of the DEGs shared by the two pairs of strong and weak hybrids at 24 DAS. **Table S15.** The results of the significantly enriched KEGG pathways of the DEGs shared by the two pairs of strong and weak hybrids at 24 DAS. **Table S16.** Total genes in the yellow module. **Table S17.** KEGG results of significant enrichment of genes in the yellow module. **Table S18.** Hub genes in the yellow module. **Table S19.** GO terms that were significantly enriched with respect to hub genes in the yellow module. **Table S20.** Specific information about the sequence of the quantitative primers of randomly selected genes.

## Data Availability

These sequence data reported in this paper have been deposited in the Genome Sequence Archive (https://ngdc.cncb.ac.cn/gsa/) with accession number CRA005745.

## Abbreviations

DEGs: Differentially expressed genes
DAS: Days after sowing
TPM: Transcripts per million
FC: Fold change
FDR: False discovery rate
GO: Gene Ontology
KEGG: Kyoto Encyclopedia of Genes and Genomes
IAA: Indole-3-acetic acid
SAUR: Small auxin-upregulated RNA
B-ARR: Type B-Arabidopsis response regulator
A-ARR: Type A-Arabidopsis response regulator
MPV: Mid-parent value
LHC: Light-harvesting complex
PSII: Photosystem II
PSI: Photosystem I
WGCNA: Weighted gene co-expression network analysis
qRT-PCR: Quantitative real-time polymerase chain reaction
