# Supplementary material for: Comparative transcriptomic analysis reveals the molecular mechanism underlying seedling biomass heterosis in Brassica napus
Source: BMC Plant Biol. 2022 Jun 9;22:283. doi: 10.1186/s12870-022-03671-0 (PMC9178846; doi:10.1186/s12870-022-03671-0)
Supplement: Supplementary file 1 — Additional file 1: Figure S1. Photos showing the seedling phenotypes of the parents and hybrids in the T1 and T2 periods.T1: 21DAS; T2: 24 DAS; Male parent: A, C, H; Female parent: M, W; Hybrid F1:AM, CM, HM, HW. Figure S2. Pearson correlation heat map between all RNA sequencing samples. The red color indicates a higher correlation between samples. -1, -2, and -3 represent three biological replicates at each time point. T1: 21 DAS; T2: 24DAS; -l: leaf. Figure S3. Number of the expression level of dominant(ELD) genes in hybrid canola seedlings in each of the 12 DEG types in the T1 and T2 periods. Genes with an expression degree in the F1 hybrids analogous to that of the female parent are defined as ELD-F; genes with an expression degree in the F1 hybrids analogous to that of the male parent are defined as ELD-M. T1: 21 DAS;T2: 24 DAS. Figure S4. Venn diagram analysis of the significant molecular function (MF) and cellular composition (CC) of the parental-ELD gene at 21 DAS. A and B display Venn diagrams of MF and CC, respectively. MF: molecular function; CC: cell component Figure S5. Venn diagram analysis of the significant molecular function (MF) andcellular composition (CC) of the parental-ELD gene at 24 DAS. A and B show Venn diagrams of MF and CC, respectively. MF: molecular function; CC: cell component. Figure S6. Remarkable biological processes of the parental-ELD gene at T1 and T2 in different hybrids.A and B exhibit Venn diagrams of the parental-ELD with significantly enriched BP in theT1 and T2 periods, respectively. C and D show heat maps of the BP terms where the parental-ELD gene is significantly enriched in different hybrids at 21 DAS and 24 DAS, respectively. T1: 21 DAS; T2: 24 DAS. Figure S7. Analysis of differentially expressed genes between strong and weak hybrids at 24 DAS.A Venn diagram of the number of unique and shared DEGs between the two groups of strong and weak hybrids at 24 DAS. B GO terms with significant enrichment of DEGs shared between t [file 12870_2022_3671_MOESM1_ESM.docx]

**Title Page:**

**Comparative transcriptomic analysis reveals the molecular mechanism underlying seedling biomass heterosis in *Brassica napus***

Jie Xiong^1^, Kaining Hu^1^, Nesma Shalby^1^, Chenjian Zhuo^1^, Jing Wen^1^, Bin Yi^1^, Jinxiong Shen^1^, Chaozhi Ma^1^, Tingdong Fu^1^, Jinxing Tu^1^*

^1^National Key Laboratory of Crop Genetic Improvement, Hubei Hongshan Laboratory, Huazhong Agricultural University, Wuhan 430070, China.

*** Corresponding author:** Jinxing Tu, E-mail: [tujx@mail.hzau.edu.cn](mailto:tujx@mail.hzau.edu.cn)


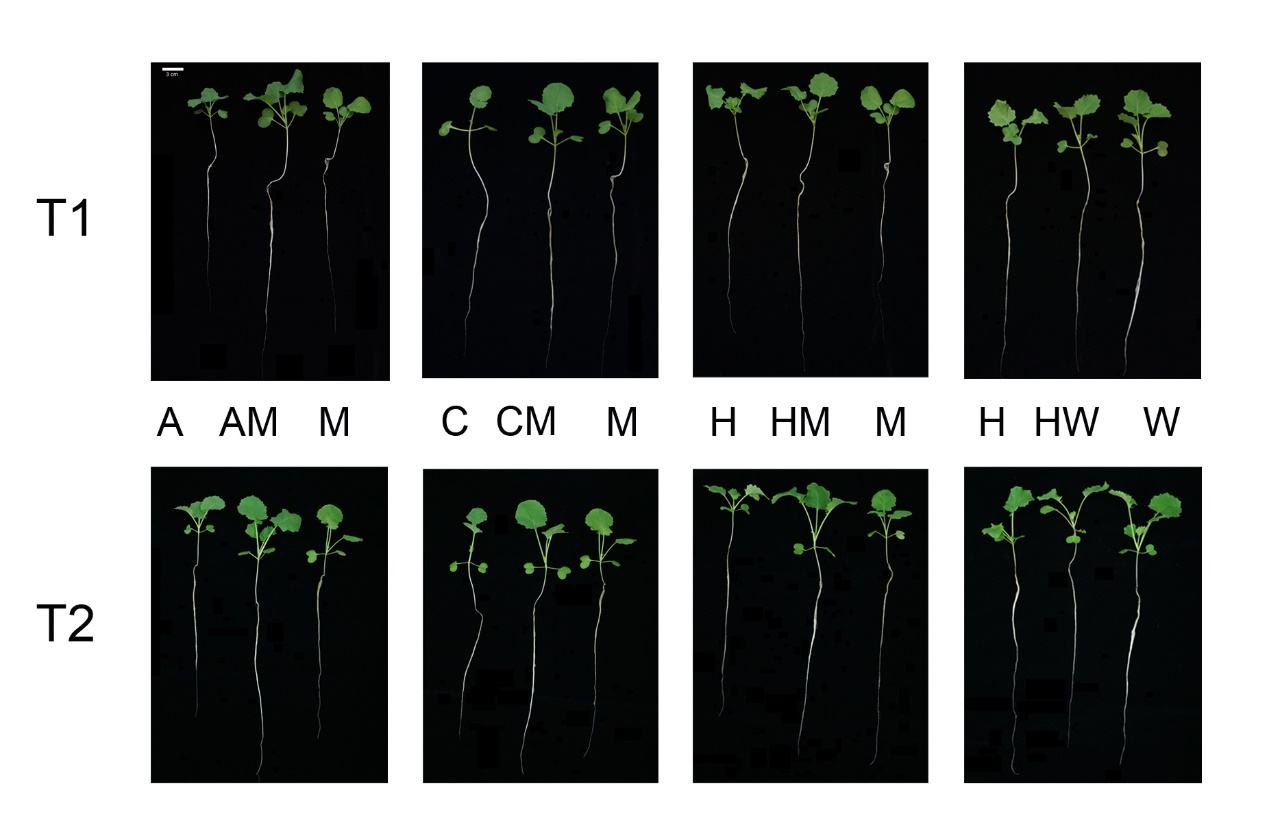


**Figure S1.** **Photos showing the seedling phenotypes of the parents and hybrids in the T1 and T2 periods.**

T1: 21 DAS; T2: 24 DAS; Male parent: A, C, H; Female parent: M, W; Hybrid F_1_: AM, CM, HM, HW.


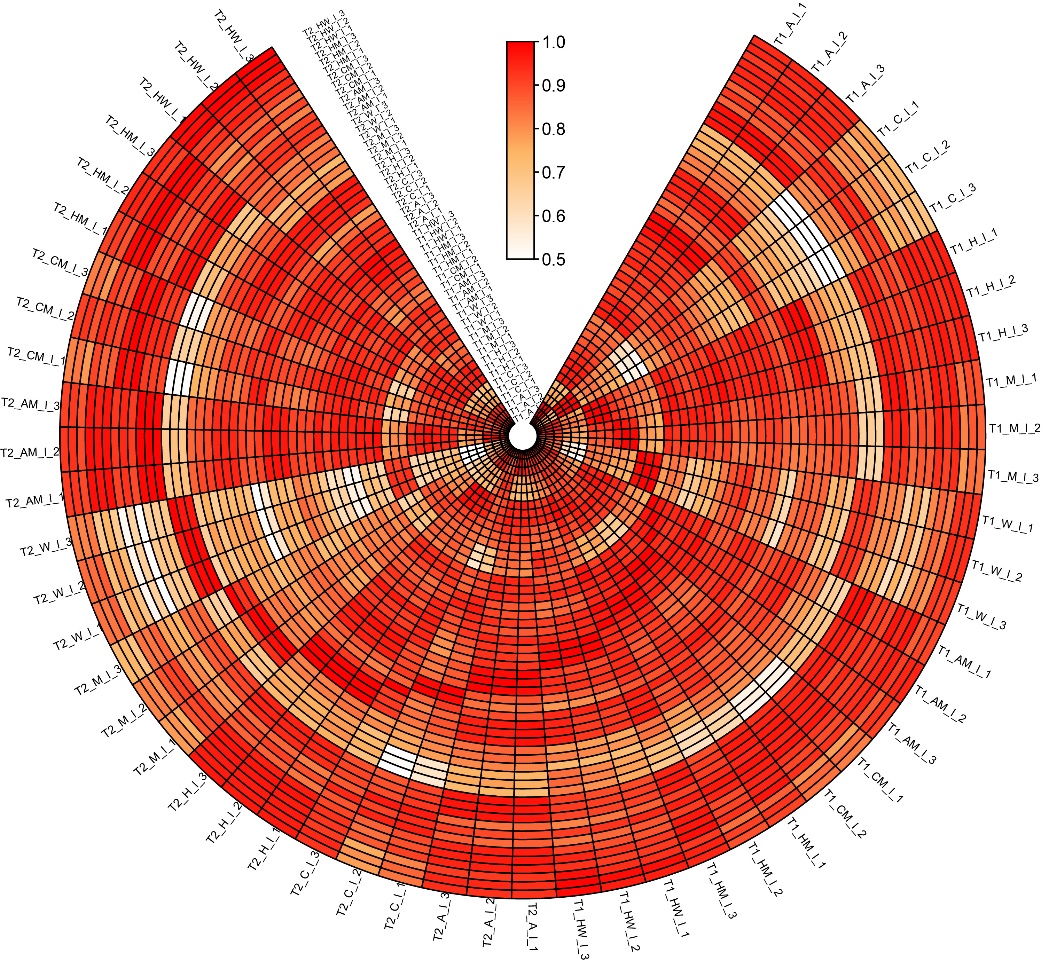


**Figure S2.** **Pearson correlation heat map between all RNA sequencing samples.**

The red color indicates a higher correlation between samples. -1, -2, and -3 represent three biological replicates at each time point. T1: 21 DAS; T2: 24 DAS; -l: leaf.


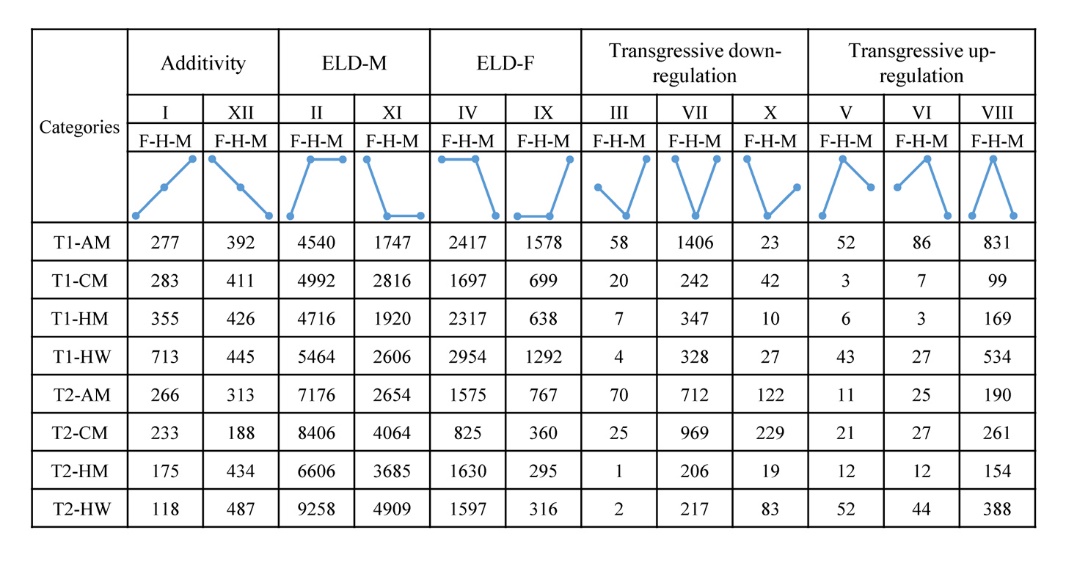


**Figure S3.** **Number of the expression level of dominant (ELD) genes in hybrid canola seedlings in each of the 12 DEG types in the T1 and T2 periods.**

Genes with an expression degree in the F_1_ hybrids analogous to that of the female parent are defined as ELD-F; genes with an expression degree in the F1 hybrids analogous to that of the male parent are defined as ELD-M. T1: 21 DAS; T2: 24 DAS.


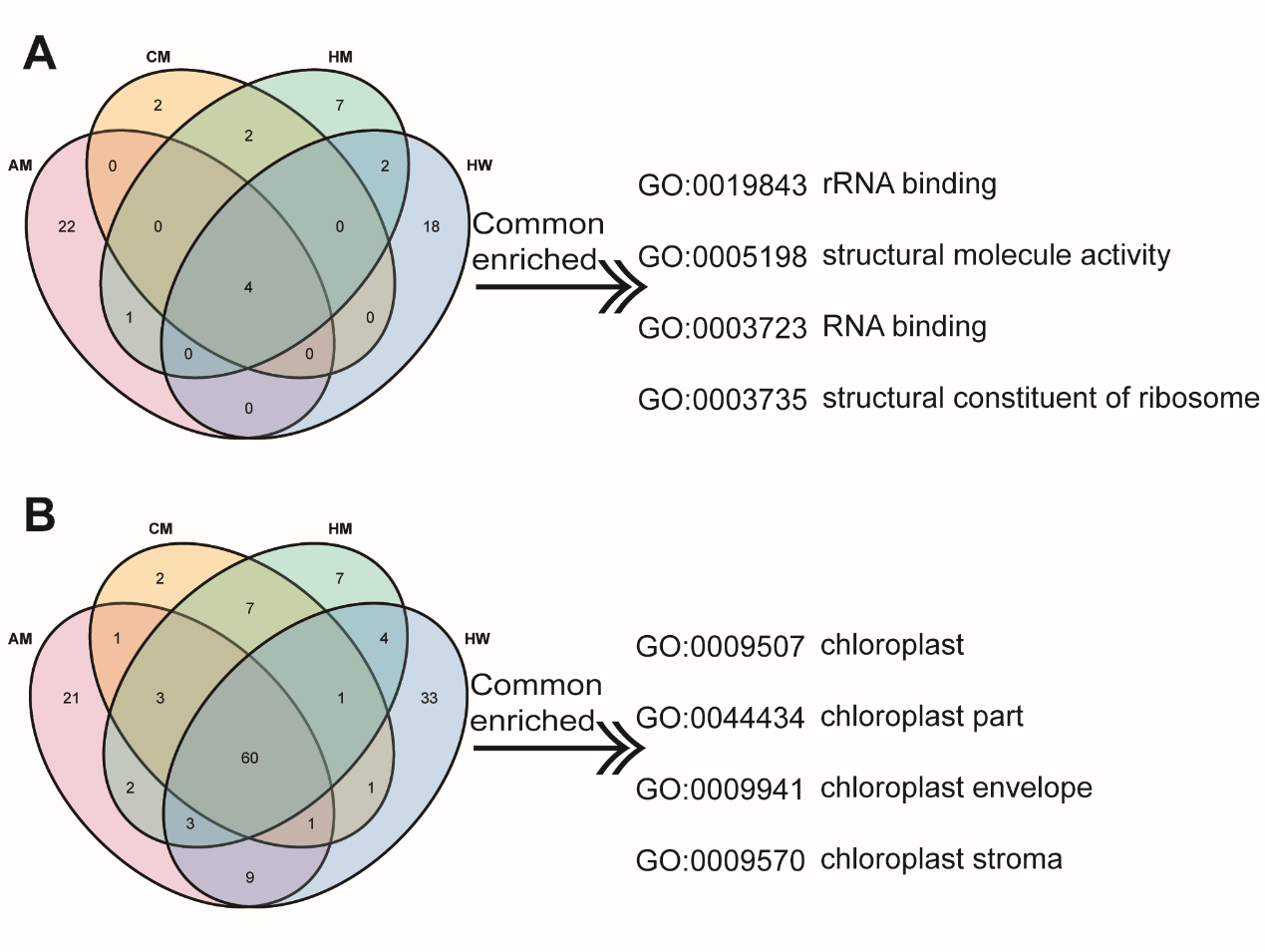


**Figure S4.** **Venn diagram analysis of the significant molecular function (MF) and cellular composition (CC) of the parental-ELD gene at 21 DAS.**

**(A)** and **(B)** display Venn diagrams of MF and CC, respectively. MF: molecular function; CC: cell component.


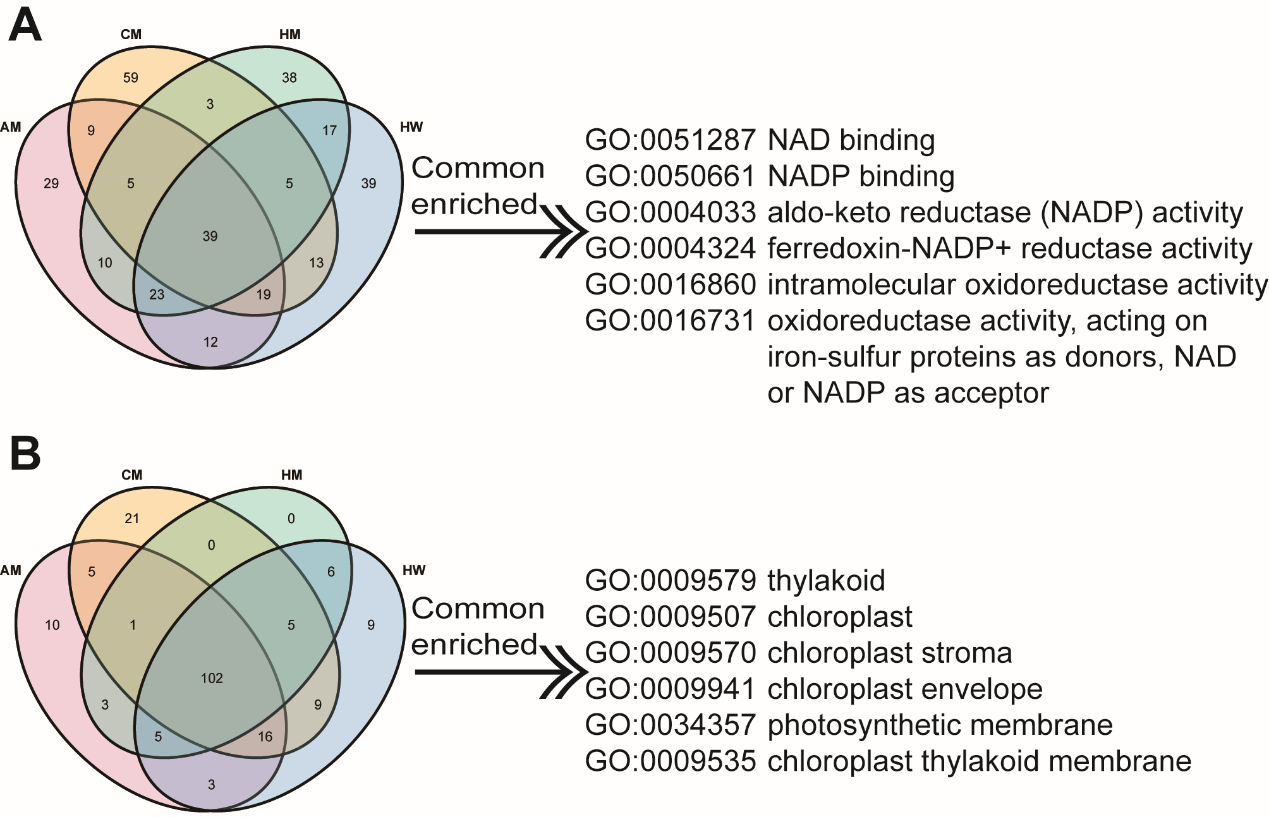


**Figure S5.** **Venn diagram analysis of the significant molecular function (MF) and cellular composition (CC) of the parental-ELD gene at 24 DAS.**

**(A)** and **(B)** show Venn diagrams of MF and CC, respectively. MF: molecular function; CC: cell component.


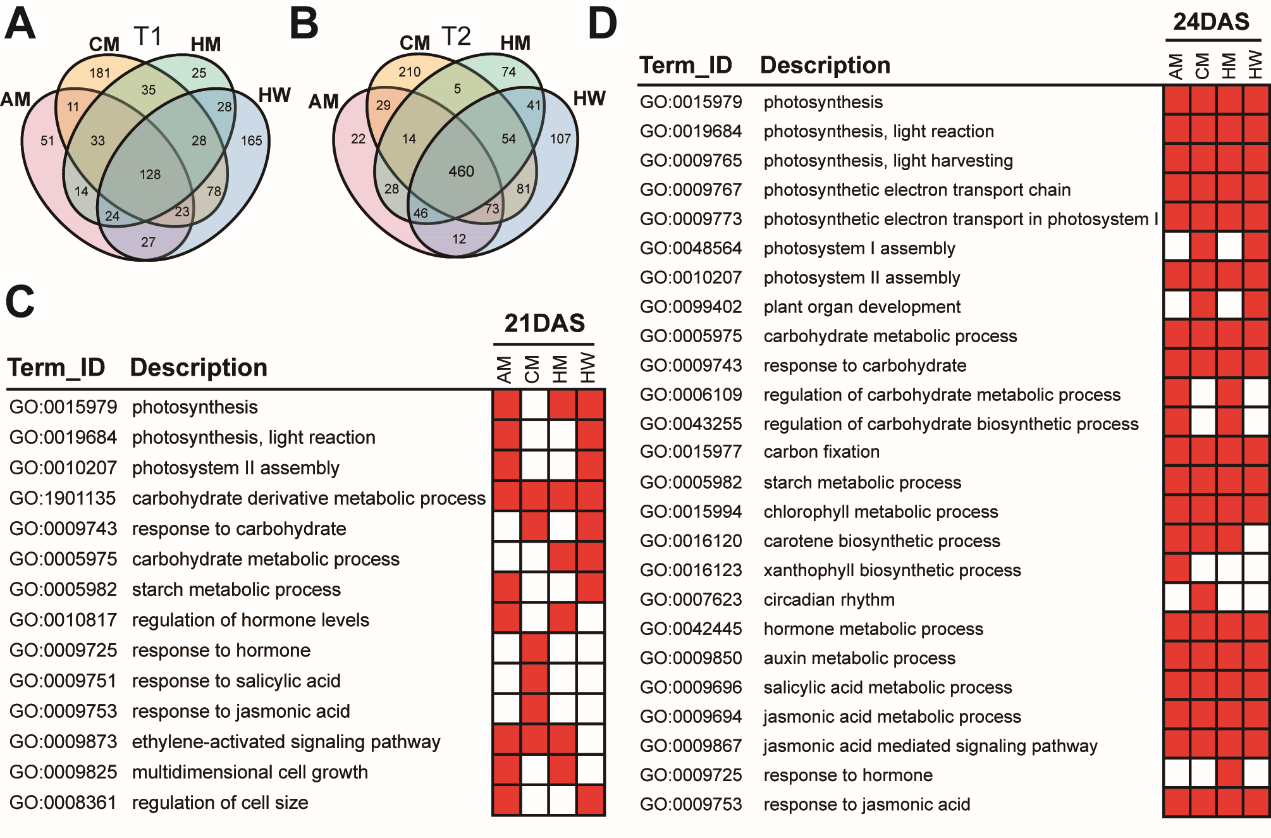


**Figure S6.** **Remarkable biological processes of the parental-ELD gene at T1 and T2 in different hybrids.**

**(A)** and **(B)** exhibit Venn diagrams of the parental-ELD with significantly enriched BP in the T1 and T2 periods, respectively. **(C)** and **(D)** show heat maps of the BP terms where the parental-ELD gene is significantly enriched in different hybrids at 21 DAS and 24 DAS, respectively. T1: 21 DAS; T2: 24 DAS.


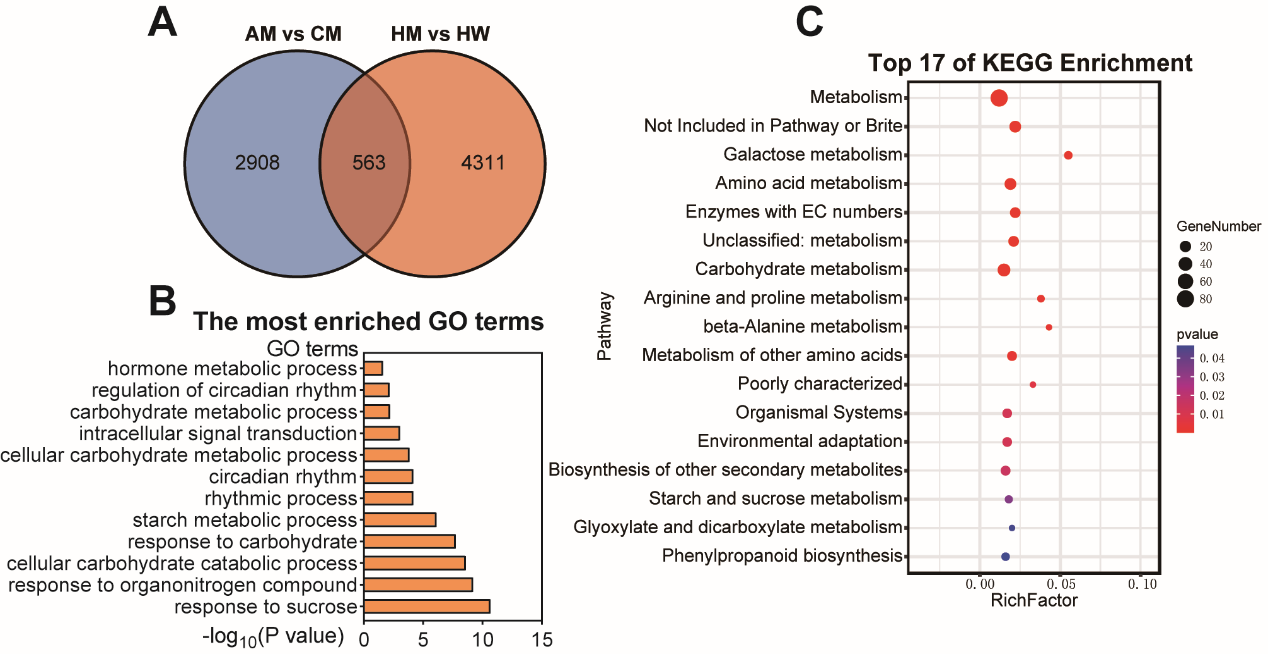


**Figure S7.** **Analysis of differentially expressed genes between strong and weak hybrids at 24 DAS.**

**(A)** Venn diagram of the number of unique and shared DEGs between the two groups of strong and weak hybrids at 24 DAS. **(B)** GO terms with significant enrichment of DEGs shared between the two strong and weak hybrids at 24 DAS. **(C)** The significantly enriched KEGG terms of DEGs shared between the two strong and weak hybrids at 24 DAS.


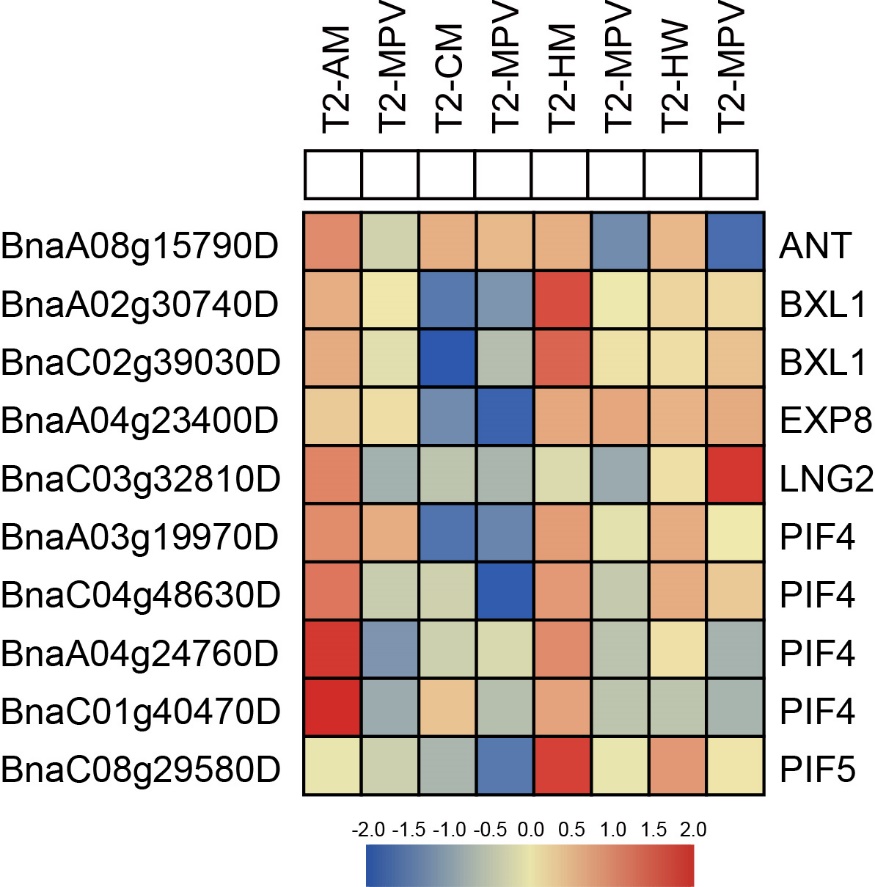


**Figure S8**. **Heat map of genes that promoted cell size in F_1_ hybrids relative to their parents at 24 DAS.**

T2: 24 DAS.


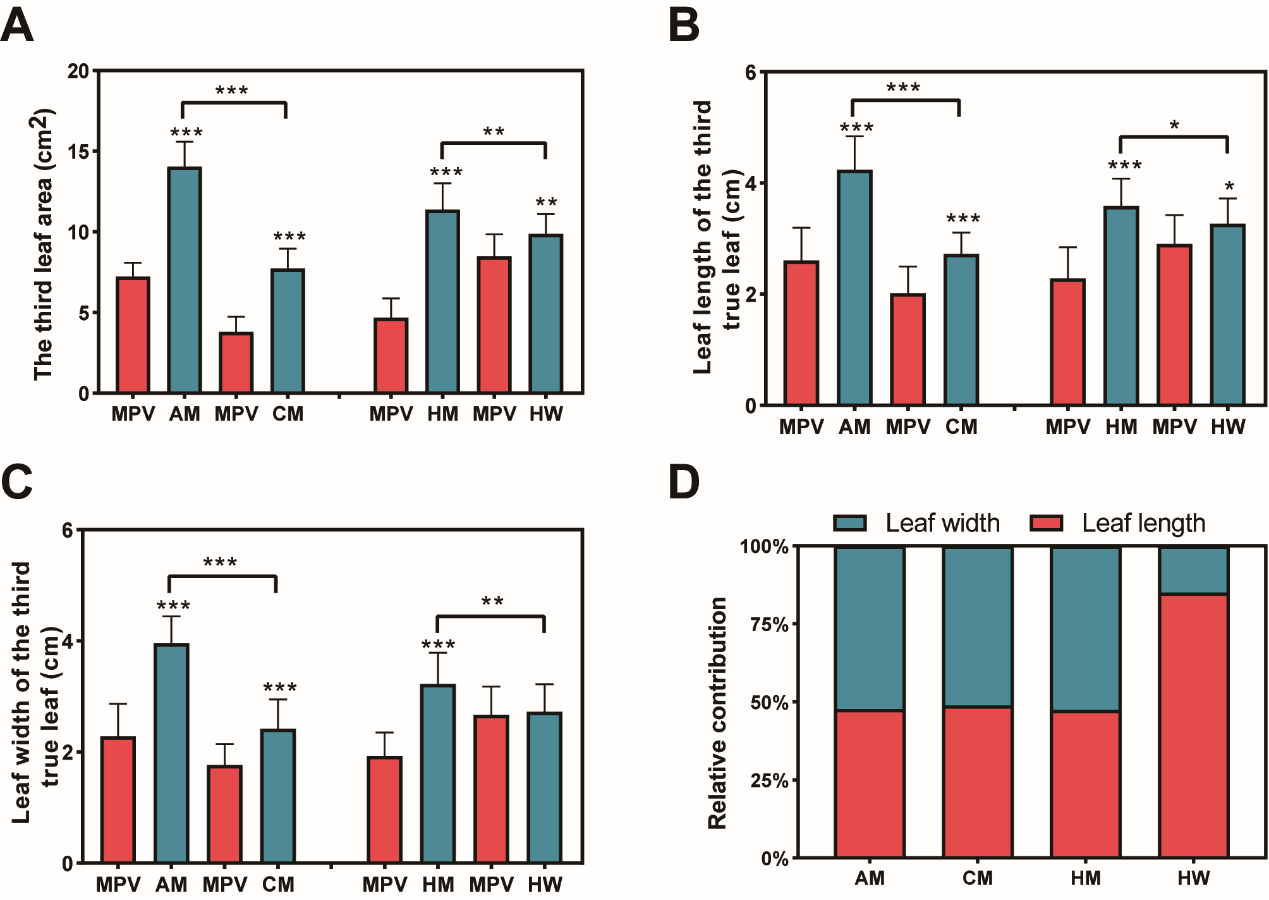


**Figure S9.** **Phenotypic changes of the leaf area of the third true leaf of all hybrids relative to their parents at 24 DAS.**

The third true leaf area **(A)**, leaf length **(B)**, and leaf width **(C)** of all F_1_ hybrids and their MPV at 24 DAS. **(D)**, The histogram shows the percentage of the contribution of leaf length and leaf width to the third true leaf area in four hybrids at 24 DAS. The data are expressed as mean ± SD, derived from the results of three biological replicates. AM, CM, HM, and HW are F_1_ hybrids; MPV: mid-parent value; *P<0.05; **P<0.01; ***P<0.001.


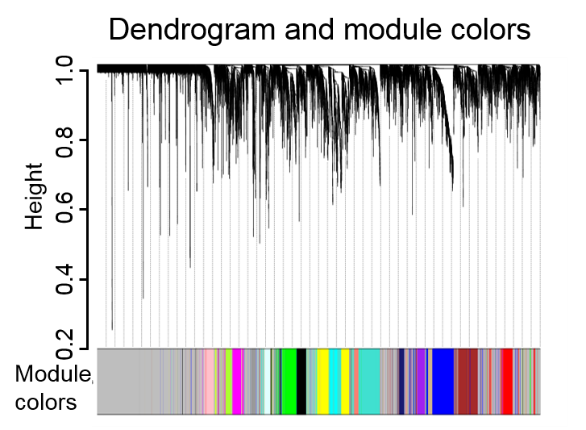


**Figure S10.** **Hierarchical clustering tree of 17 modules obtained by WGCNA.**


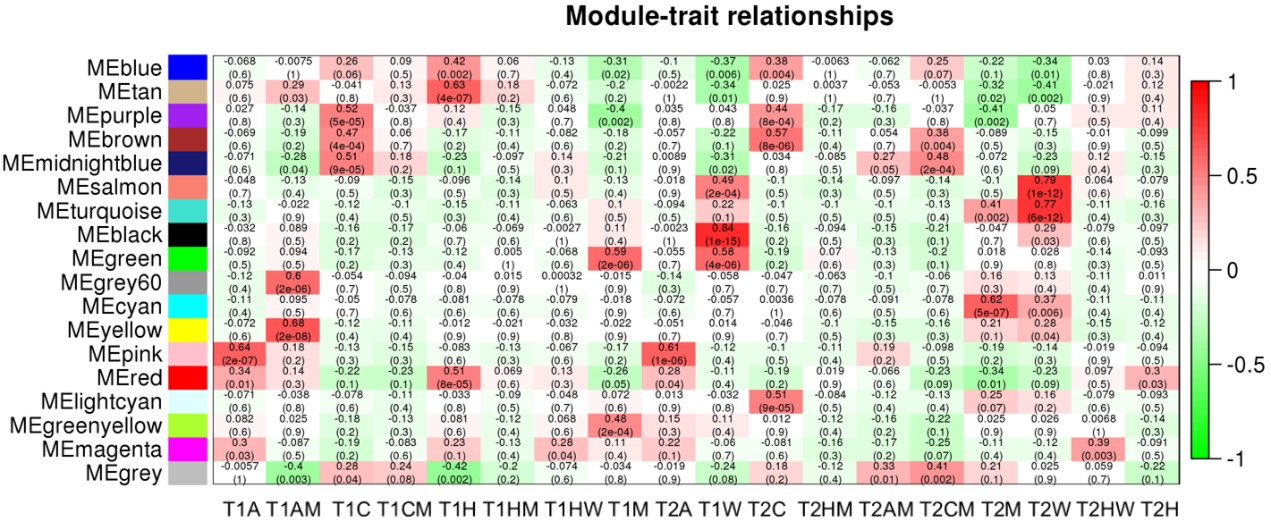


**Figure S11.** **Heatmap of the correlation analysis of modular traits.** Each row represents a module and each column represents a different sample. The correlation coefficients and P-values are shown in the figure. Red and green represent positive and negative correlations, respectively.


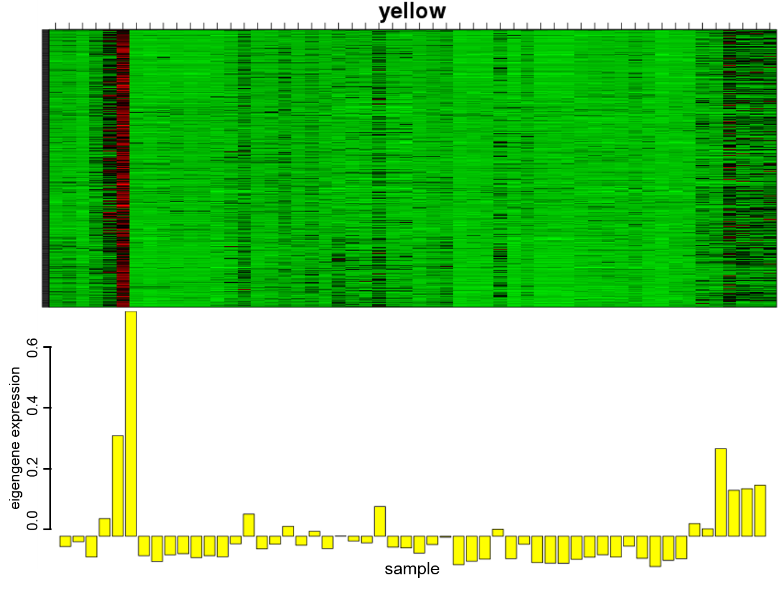


**Figure S12.** **Heatmap showing the eigengene expression profile of the yellow module in different samples.**


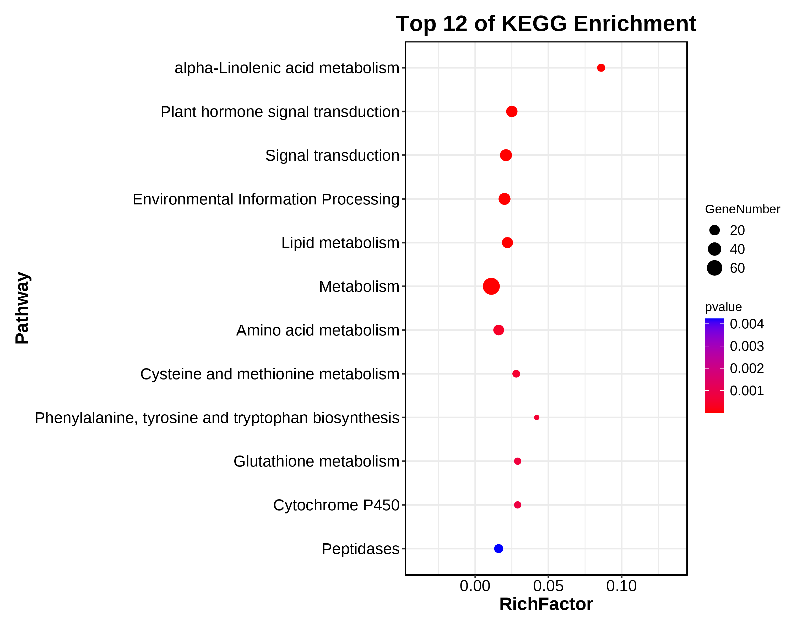


**Figure S13.** **Significantly enriched KEGG terms in yellow modules.**


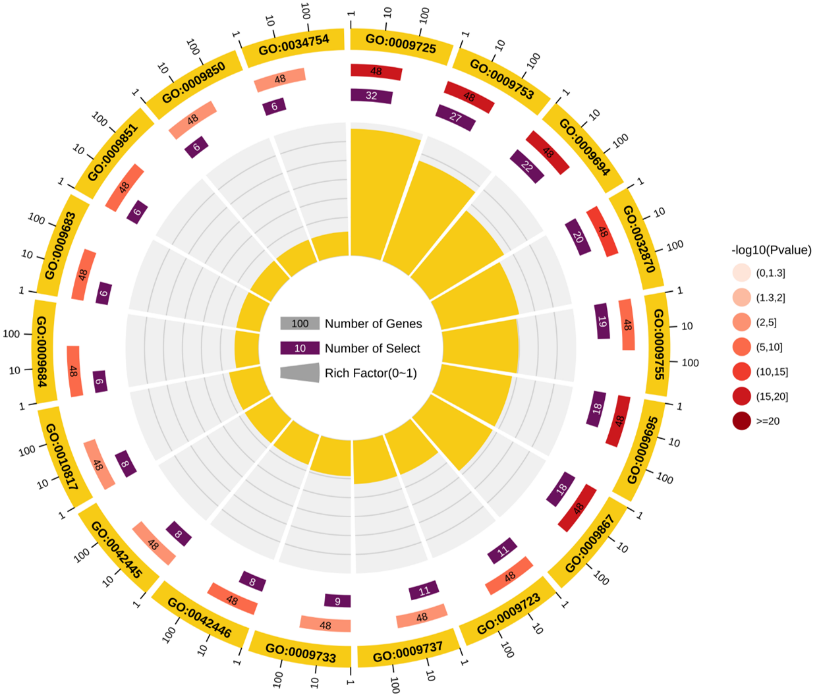


**Figure S14.** **Circle chart of GO terms showing significant enrichment of hub genes in the yellow module.** The first circle is the enriched classification, and the outer circle is the coordinate ruler of the number of genes. The second circle is the number of the category in the background gene. The third circle is the number enriched in the category. The fourth circle is the value of the rich factor for each category.


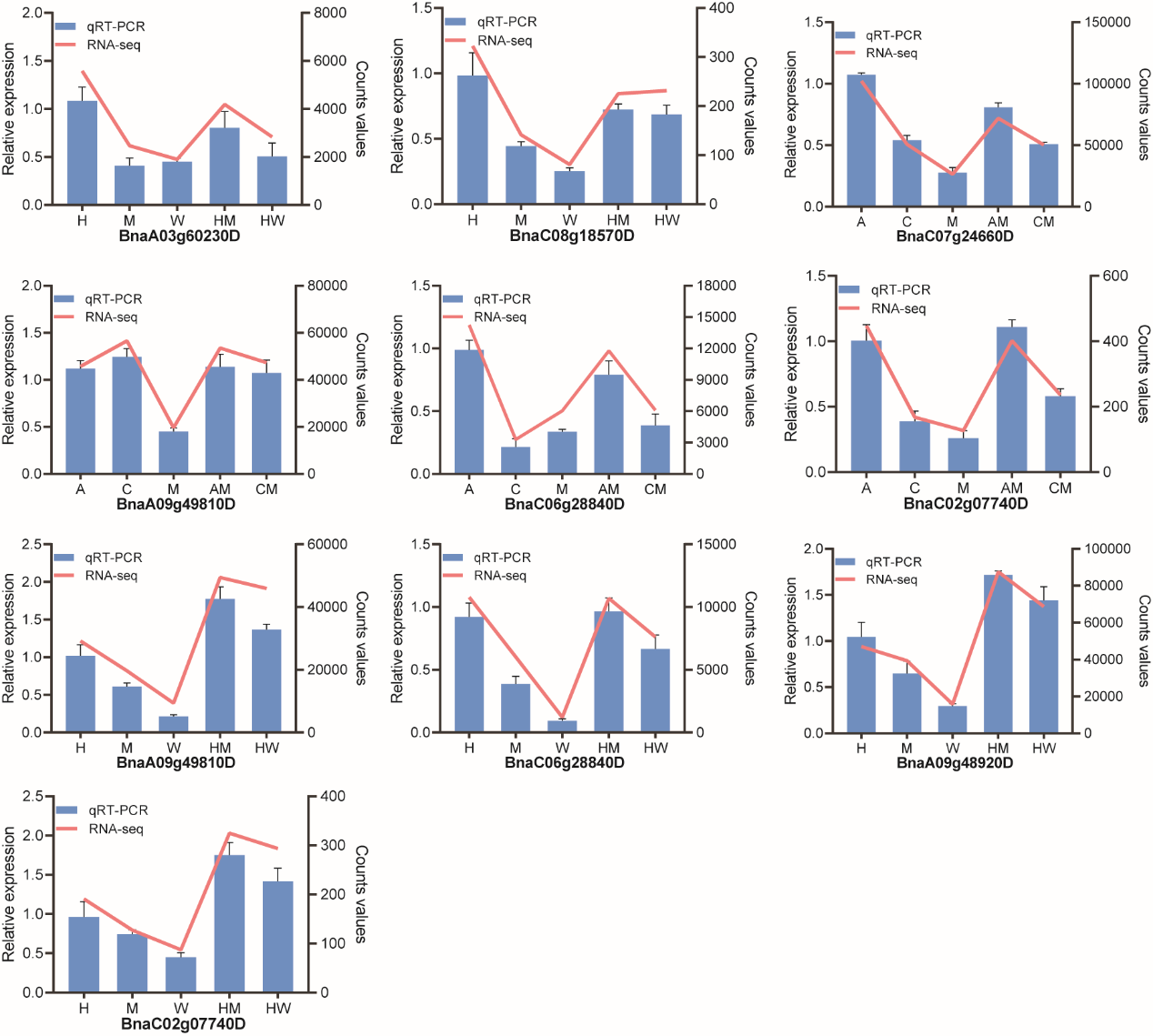


**Figure S15.** **qRT-PCR validation of the expression degrees of 10 genes in the photosynthetic and plant hormone signal transduction pathways.**

The blue histogram shows the relative expression levels (mean ± SD) of genes obtained through quantitative verification. The red line graph depicts the gene expression level value obtained by the transcriptome. Male: A, C, H; Female: M, W; F_1_ hybrid: AM, CM, HM, HW. The first four genes were expressed at 21 DAS, whereas the other genes were expressed at 24 DAS.


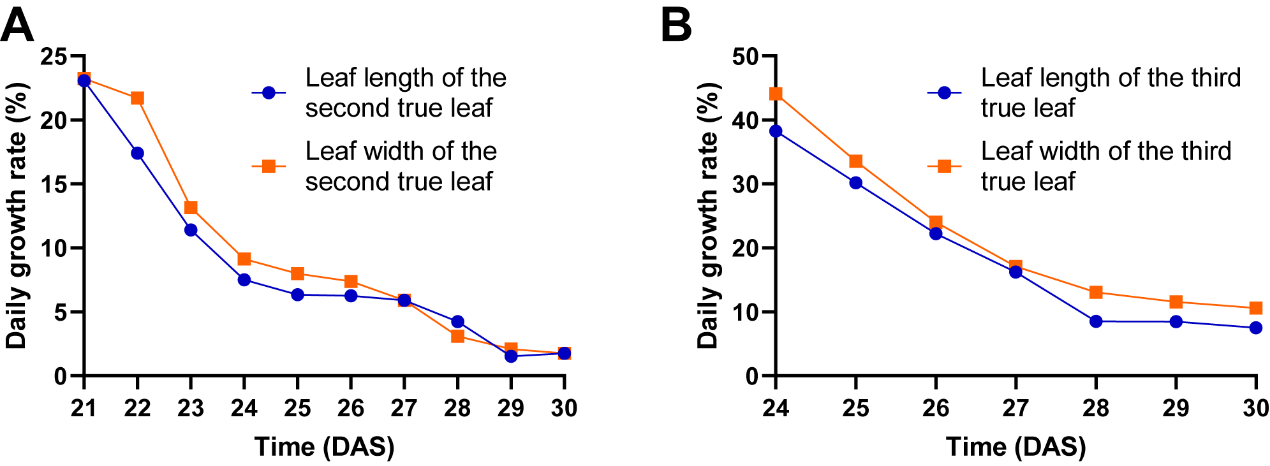


**Figure S16.** **Determination of the leaf sampling period.**

Figures **(A)** and **(B)** represent the line graphs of the measured daily growth rate of the leaf length and breadth of the second and third true leaves, respectively, at 20–30 DAS.
